# Piezo1 Regulates Odontogenesis via a FAM83G-Mediated Mechanism in Dental Papilla Cells In Vitro and In Vivo

**DOI:** 10.3390/biom15030316

**Published:** 2025-02-20

**Authors:** Xinyue Sheng, Jingzhou Li, Haozhen Ma, Hongwen He, Qin Liu, Shilin Jia, Fuping Zhang, Fang Huang

**Affiliations:** 1 Hospital of Stomatology, Sun Yat-sen University, Guangzhou 510055, China; shengxy@mail2.sysu.edu.cn (X.S.); lijzh5@mail2.sysu.edu.cn (J.L.); mahzh5@mail2.sysu.edu.cn (H.M.); hehw@mail.sysu.edu.cn (H.H.); liuq95@mail3.sysu.edu.cn (Q.L.); jiashlin@mail2.sysu.edu.cn (S.J.); 2 Guangdong Provincial Key Laboratory of Stomatology, Guangzhou 510080, China

**Keywords:** Piezo1, odontogenic differentiation, dental papilla cells, FAM83G, Yoda1

## Abstract

This study explored the role of Piezo1 in the odontogenic differentiation of dental papilla cells (DPCs) and tissue, focusing on a mechanism involving family with sequence similarity 83, member G (FAM83G). Here, we found Piezo1, a mechanosensitive cation channel, was upregulated during odontogenesis in DPCs and dental papilla tissues. Knockdown of Piezo1 impaired odontogenic differentiation, while its activation by Yoda1 enhanced the process. Using a 3D culture model and an ectopic transplantation model, we confirmed Piezo1’s role in vivo. RNA sequencing (RNA-seq) analysis revealed that FAM83G was upregulated in Piezo1-knockdown cells, and FAM83G silencing enhanced odontogenesis in DPCs. These findings indicate that Piezo1 positively regulates odontogenesis by inhibiting FAM83G in DPCs both in vitro and in vivo, with Piezo1 representing a potential target for dental tissue regeneration.

## 1. Introduction

Tooth development is a complex and intricately regulated biological process, closely associated with mechanical stimulation [1,2]. Existing clinical and laboratory evidence suggests that orthodontic forces have a promotive effect on dentin formation in immature permanent teeth [1,3]. Abnormal mechanical stimuli can result in dental development anomalies or pathological conditions [4]. In the tooth germ, the dental papilla serves as the source of both dental pulp and dentin and plays a crucial role in determining the shape and structure of the tooth [5,6]. As tooth-derived mesenchymal stem cells (MSCs), dental papilla cells (DPCs) possess the potential for multi-directional differentiation, making them ideal seed cells for the study of tooth development and dental tissue engineering. The DPCs adjacent to the inner enamel epithelium differentiate first into odontoblasts, while the inner DPCs eventually develop into dental pulp cells. Given the critical role of DPCs in tooth development, we hypothesize that extracellular mechanical stimuli in the environment may influence their differentiation fate through specific mechanisms.

Piezo1 is a non-selective mechanosensitive cation channel discovered in 2010, characterized by its unique “triple propeller” structure that endows it with the ability to sense mechanical stimuli [7,8,9]. It can sense and transduce extracellular mechanical signals [9,10,11], including membrane tension [12], membrane curvature [13], hydrostatic pressure [14], fluid shear stress [15], and substrate stiffness [16,17]. These signals are converted into biochemical signals recognizable by cells, thereby regulating cellular behavior and differentiation fate [14,18]. Existing research has shown that Piezo1 plays a crucial role in various dental-derived stem cells. For instance, in periodontal ligament stem cells, the activation of Piezo1 promotes osteogenic differentiation and contributes to the reconstruction of periodontal tissues during orthodontic tooth movement (OTM) [19]. Additionally, Piezo1 is involved in the odontogenic differentiation and mineralization of dental pulp stem cells and stem cells from human exfoliated deciduous teeth, influencing key processes such as osteogenic/adipogenic differentiation and cell proliferation [20,21,22,23]. Despite the insights gained into Piezo1’s roles in these cells, it remains unclear whether Piezo1 participates in dentin formation during tooth development, and even less is known about its role in the odontogenic differentiation of DPCs.

Therefore, we hypothesize that Piezo1 can regulate the odontogenic differentiation of DPCs and dentin formation both in vitro and in vivo. To test this hypothesis and further explore the molecular mechanisms, we employed RNA sequencing (RNA-seq) technology and revealed its downstream signaling pathway. Our findings suggest that Piezo1 may exert its effects through a downstream molecule, namely, family with sequence similarity 83, member G (FAM83G). This study not only broadens the theoretical understanding of tooth development and OTM in immature permanent teeth, but also identifies Piezo1 as a potential new target for dental tissue regeneration.

## 2. Materials and Methods

### 2.1. Immunohistochemistry (IHC)

This experiment was approved by the Institutional Animal Care and Use Committee, Sun Yat-sen University, Guangzhou, China (No. SYSU-IACUC-2023-001546). Sprague-Dawley (SD) rats aged 3–5 days postnatal were sourced from the Laboratory Animal Center, Sun Yat-sen University. The rats were euthanized with an overdose of anesthesia, and bilateral mandibles were extracted for tissue sampling. The tissues were fixed in 4% paraformaldehyde at room temperature for 24 h, followed by immersion in 10% EDTA decalcification solution for 7 days with agitation. After gradient dehydration and clearing, the specimens were embedded in paraffin and sectioned consecutively into 4 μm slices. For IHC, the sections were dewaxed, rehydrated, subjected to antigen retrieval via EDTA microwave treatment, and blocked with BSA before overnight incubation with anti-Piezo1 antibody (Table 1) at 4 °C. On the following day, sections were treated with HRP-conjugated secondary antibodies at room temperature. This was followed by DAB staining and hematoxylin counterstaining for 3 min. After dehydration and clearing with a gradient ethanol, propanol, and xylene, the sections were mounted and analyzed using a digital slide scanner.

### 2.2. Cell Isolation, Culture, and Differentiation

SD rats aged 1–2 days postnatal were sourced from the Laboratory Animal Center, Sun Yat-sen University. Dental papilla tissues were isolated and digested following established protocols [24,25]. The treated tissues and dispersed cells were cultured in α-MEM (Gibco, Grand Island, NY, USA) supplemented with 15% fetal bovine serum (FBS, Cyagen, Santa Clara, CA, USA). When cells reached 80–90% confluency, they were passaged and purified using differential digestion with TrypLE (Gibco). Subsequently, the cells were cultured in α-MEM supplemented with 8% FBS, 100 μg/mL glutamine (Gibco), 100 U/mL penicillin, and 100 μg/mL streptomycin (Gibco). Cells from passages 2–4 were utilized for subsequent experimental procedures.

For odontogenesis, DPCs were cultured in osteo/odontogenic induction (OS) medium, comprising α-MEM supplemented with 6% FBS, 100 μg/mL glutamine, 100 U/mL penicillin, 100 μg/mL streptomycin, 10 mM β-glycerophosphate, 0.2 mM vitamin C, and 10 nM dexamethasone.

For adipogenic differentiation, DPCs were cultured in α-MEM supplemented with 8% FBS, 100 μg/mL glutamine, 100 U/mL penicillin, 100 μg/mL streptomycin, 1 μM dexamethasone, 200 μM indomethacin, 0.5 mM 3-isobutyl-1-methylxanthine, and 10 μg/mL insulin. After 21 days of culture, cells were stained with Oil Red O solution (Cyagen) and observed under a microscope for imaging.

### 2.3. Semi-Quantitative Alizarin Red Staining (ARS)

Cells were initially fixed with 4% paraformaldehyde, followed by staining with Alizarin Red S Solution (Cyagen) in the dark. Following rinsing with double distilled water, the plates were scanned using a scanner, and observations and photographs were captured under an inverted microscope. For semi-quantitative analysis, the samples were fully dissolved using 100 mM cetylpyridinium chloride (Sigma-Aldrich, St. Louis, MO, USA). Subsequently, the optical density (OD) values of each group at a wavelength of 562 nm were measured in the collected supernatant using a spectrophotometer.

### 2.4. Cell Clonogenic Assay

Healthy DPCs were seeded at a very low density (approximately 1000 cells) in a 6 cm^2^ cell culture dish and cultured for 14 days for clonogenic assays. The cells were rinsed and stained with 0.1% crystal violet solution at room temperature for 20 min. After washing with PBS, cells were observed and photographed under an inverted microscope.

### 2.5. Flow Cytometry

To identify MSCs, DPCs in optimal condition were processed into single-cell suspensions. After filtration through a 70 μm mesh and centrifugation at 1000 rpm for 5 min to remove the supernatant, the cell density of the suspension was adjusted to 5 × 10^6^ cells/mL with PBS containing 1% BSA. Antibodies conjugated with FITC for CD44, CD45, and CD90, along with antibodies conjugated with PE for CD29 and CD34, were added individually to the suspension, along with their isotype controls (Elabscience, Wuhan, Hubei, China). After gentle vortexing, the mixtures were incubated at room temperature in the dark for 30 min. After washing and diluting with PBS, the suspension was thoroughly resuspended and analyzed using a flow cytometer.

### 2.6. Immunofluorescence (IF)

For immunofluorescence analysis, cells in optimal condition were seeded at a density of 2 × 10^3^ cells per 35 mm^2^ laser confocal dish. The cells were then rinsed with PBS, fixed with 4% paraformaldehyde, permeabilized with 0.1% Triton-100, and blocked with 5% BSA. Subsequently, the cells were incubated overnight at 4 °C with one of the following antibodies: anti-Cytokeratin, anti-Vimentin, or anti-Piezo1 antibody (Table 1). The WSU-HN6 cell line was established from the oral squamous cell carcinoma of a male patient and serves as a positive control for Cytokeratin due to its epithelial origin [24,26]. On the next day, DyLight 488/594-conjugated secondary antibodies were added to each group under dark conditions. Finally, the cells were counterstained with DAPI-containing antifade mounting medium (Beyotime, Shanghai, China) and observed and photographed using a laser confocal microscope.

### 2.7. Real-Time Quantitative Polymerase Chain Reaction (RT-qPCR)

To quantify the mRNA expression levels of the target genes, the following experimental procedures were conducted in accordance with the instructions provided with each kit. Total RNA from cells was extracted (Yishan Biotechnology, Shanghai, China), followed by genomic DNA removal and cDNA synthesis (Yeasen, Shanghai, China). Subsequently, reaction mixtures were prepared, consisting of the aforementioned cDNA, SYBR Green (Yeasen), and primers (RuiBiotech, Guangzhou, Guangdong, China; sequences listed in Table 2). RT-qPCR analysis was performed using the Roche LightCycler 96 instrument, following the recommended protocol. Internal normalization was performed with β-actin (*Actb*), and the comparative expression of the gene of interest was calculated using the 2^−ΔΔCt^ method.

### 2.8. Western Blotting (WB)

To quantify the protein expression levels of the target genes, total protein was extracted from cells at 4 °C using radioimmunoprecipitation assay (RIPA) lysis buffer (Beyotime) containing 1% phenylmethylsulfonyl fluoride (PMSF, Beyotime). Protein concentrations were measured with a bicinchoninic acid (BCA) assay kit (CWBio, Beijing, China). The protein samples were mixed with 5× Loading Buffer (CWBio) and denatured. Electrophoresis was performed on 4–12% SDS-PAGE precast gels (ACE Biotechnology, Changzhou, Jiangsu, China), followed by transfer onto polyvinylidene fluoride (PVDF) membranes (Millipore, Billerica, MA, USA). The membranes were blocked in skim milk at room temperature for 2 h and incubated with antibodies overnight at 4 °C. Then, the membranes were incubated with corresponding HRP-conjugated secondary antibodies at room temperature for 1 h. Imaging was conducted using enhanced chemiluminescence (ECL, Millipore) with a Bio-Rad ChemiDoc chemiluminescence system, and band intensities were analyzed using ImageJ 1.53t software (NIH, Bethesda, MD, USA). Details of the antibodies are provided in Table 1. Original western blots can be found at Supplementary Materials.

### 2.9. CCK-8 Assay

After 24 h of culture in 96-well plates, DPCs in a healthy growth state were replaced with medium containing different concentrations of Yoda1 (0.1 μM, 0.5 μM, 1 μM, 5 μM, 10 μM) and further incubated for 48 h. Yoda1, a synthetic small molecule, is a commonly used specific agonist for Piezo1. It acts as a wedge to stabilize Piezo1 in an open conformation, thereby reducing the channel’s mechanical activation threshold [27,28]. Following the incubation period, cells were treated with 10% CCK-8 reagent and incubated at 37 °C for 3 h. OD values at 450 nm wavelength were then measured for each well using a spectrophotometer.

### 2.10. ALP Staining and Activity Analysis

To perform ALP staining, cells were fixed and then subjected to BCIP/NBT staining solution (Beyotime) for ALP visualization, followed by scanning and photographic documentation. For quantitative assessment of ALP activity, cellular lysates were prepared using RIPA, and protein concentrations were standardized. Subsequently, reaction working solution was added according to the instructions provided with the ALP activity assay kit (NJJCbio, Nanjing, Jiangsu, China), followed by incubation at 37 °C for 10 min. OD values at 520 nm wavelength were determined for each group using a spectrophotometer, allowing for the calculation of ALP activity within the samples.

### 2.11. Cell Transfection

To achieve knockdown of Piezo1 or FAM83G, DPCs were seeded at a density of 7.5 × 10^4^ cells per well in 12-well plates and cultured for 24 h, until they adhered to the surface and reached 50–70% confluence. The transfection process was conducted using the Lipomaster 2000 (Vayzme, Nanjing, Jiangsu, China). Specifically, 2 μL of siRNA was diluted in 62.5 μL of Opti-MEM (Gibco). Separately, 2.5 μL of the Lipomaster 2000 reagent was also diluted in 62.5 μL of Opti-MEM. Both solutions were vigorously mixed and incubated at room temperature for 5 min to form the transfection complexes. The original culture medium in the wells was replaced with fresh, antibiotic-free medium, followed by the addition of 125 μL of the transfection complex to each well in a dropwise manner. To evaluate the knockdown efficiency, total RNA and protein were extracted 48 h and 72 h post-transfection, respectively. All Piezo1- or FAM83G-targeted siRNAs (si-Piezo1/FAM83G 1, 2, and 3) and the negative control siRNA (si-NC) were designed and synthesized by GenePharma (Shanghai, China), with the sequences listed in Table 3.

### 2.12. Three-Dimensional Culture

For 3D culture, a construct was devised incorporating DPCs at a concentration of 1 × 10^7^ cells/mL within Matrigel (Corning, NY, USA), either with or without Yoda1 (1 or 5 μM). The construct was seeded into laser confocal dishes and cultured under OS condition for 5 days. Afterward, IF analysis was conducted following the aforementioned steps.

### 2.13. Ectopic Subcutaneous Transplantation Model

This experiment was approved by the Institutional Animal Care and Use Committee, Sun Yat-sen University, Guangzhou, China (No. SYSU-IACUC-2023-001762). To ascertain the impact of Piezo1 on odontogenesis by DPCs in vivo, a construct was devised incorporating DPCs at a concentration of 1 × 10^7^ cells/mL within Matrigel, either with or without Yoda1 (5 μM). Six-week-old BALB/c Nude mice, obtained from the Laboratory Animal Center, Sun Yat-sen University, were utilized for this purpose. The sample size of this experiment was determined based on preliminary experiments. Briefly, 150 μL of gel was meticulously injected into each subcutaneous cavity. The mice were euthanized using an overdose of anesthesia after a 4-week period of nurturing. Animals with severe health issues, abnormal physiological indicators, or abnormal behavior were excluded from the experiment. This experiment strictly adhered to the principle of blinding. The subcutaneous implants were extracted for subsequent histological analysis.

### 2.14. Histological Analysis

All tissue sections were dewaxed and hydrated prior to staining. For hematoxylin and eosin (H and E) staining, sections were treated with hematoxylin solution (Servicebio, Wuhan, Hubei, China) for 1 min, differentiated by immersion in 1% hydrochloric acid alcohol for 10–20 s, and rinsed under running water for 5 min for bluing. Following a rinse in double distilled water, sections were stained with eosin solution (Servicebio) for 2 min. For Masson trichrome staining, sections were stained with iron hematoxylin solution for 7.5 min, differentiated with 1% hydrochloric acid alcohol for 30 to 60 s to stain cell nuclei gray–black against a colorless or pale gray background, and then immersed in 65 °C acid fuchsin solution (Servicebio) for 6 min and 1% phosphomolybdic acid for 2 min to stain collagen fibers light red and other fibers red. The sections were further treated with aniline blue solution (Servicebio) for 5 min, followed by immersion in three containers of 1% acetic acid for 30 s each. After staining, sections were dehydrated, cleared, and mounted. The complete slides were scanned using a digital pathology scanner (Leica, Wetzlar, Hesse, Germany).

### 2.15. RNA Sequencing

The total RNA of DPCs treated with OS + si-NC and OS + si-Piezo1 for 5 days was extracted as previously stated. The mRNA sequencing and analysis were conducted by Yongnuo Biotech (Guangzhou, China). Library quality and quantity were assessed using the Agilent 2100 system and qPCR, and the libraries were sequenced on the Illumina HiSeqTM2000 platform. Each sample averaged 65 million clean reads, with raw base counts between 9.51 Gb and 9.96 Gb. Statistical analysis was performed by DESeq2. Differentially expressed genes (DEGs) were identified as fold-change >2 as well as *p*-value < 0.01. All DEGs are listed in Supplementary Table S1.

### 2.16. Statistical Analysis

Data analysis was performed using GraphPad Prism 9.0.0 (GraphPad, San Diego, CA, USA). Results from a minimum of three replicate experiments were reported as mean ± standard deviation. Two-group comparisons were made using Student’s *t* test for normal distributions or Mann–Whitney U test for non-normal distributions. Multiple group analyses were done with one-way ANOVA for normal distributions or Kruskal–Wallis H test for non-normal distributions. A *p*-value of less than 0.05 was set as the threshold for statistical significance.

## 3. Results

### 3.1. The Expression Pattern of Piezo1 in Rat Molars and DPCs

**Figure 1:**
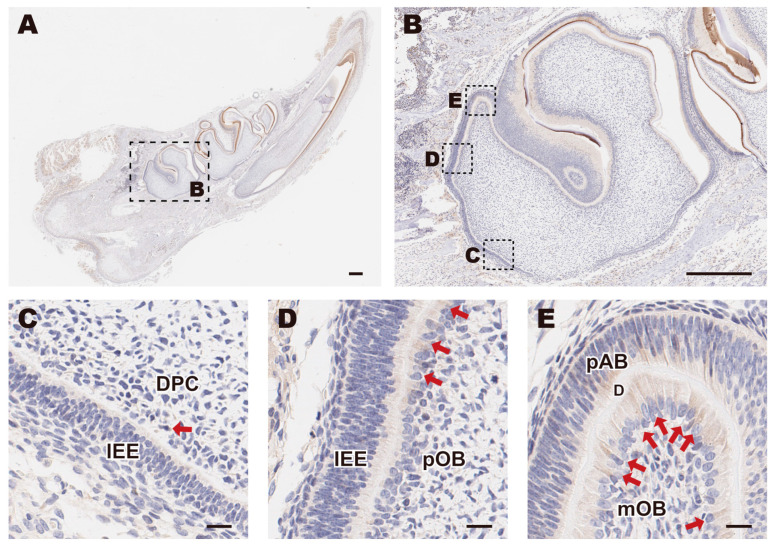
The expression pattern of Piezo1 in a rat molar. (**A**) Immunohistochemical analysis of the mandible and (**B**) its second molar from a SD rat at postnatal day 5; scale bar: 500 µm. (**C**) DPCs adjacent to the IEE underwent polarization and elongation, differentiating into (**D**) preodontoblasts, and eventually into (**E**) mature odontoblasts; scale bar: 20 µm. Red arrows point to Piezo1-positive cells. DPC, dental papilla cell; IEE, inner enamel epithelium; pOB, preodontoblast; pAB, preameloblast; mOB, mature odontoblast; D, dentin.

To investigate the expression pattern of Piezo1 during the development of dental papilla in SD rats, we selected longitudinal sections of molars containing undifferentiated DPCs to mature odontoblasts (mOBs) at various stages of differentiation for IHC staining (Figure 1A,B). Piezo1 expression was nearly undetectable in DPCs located near the root of the molar (Figure 1C). However, the number of Piezo1-positive cells gradually increased in preodontoblasts (pOBs) (Figure 1D) and reached a maximum in mOBs (Figure 1E).

**Figure 2:**
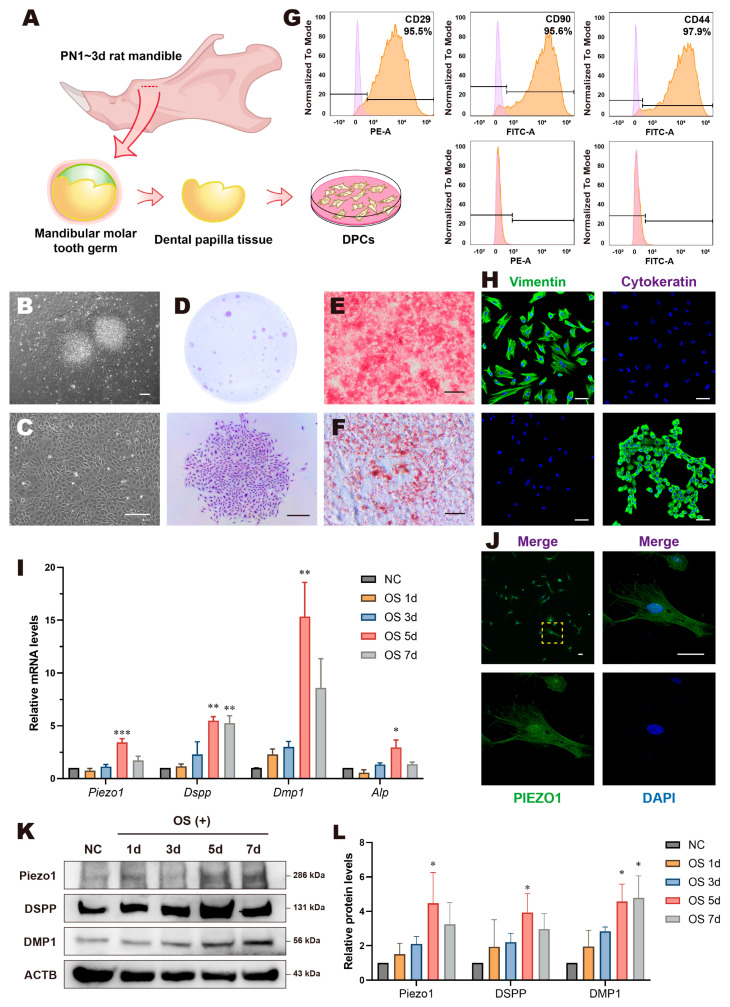
DPC identification and the expression pattern of Piezo1 in vitro. (**A**) Schematic model of DPC extraction from SD rat mandible molars. (**B**) Primary cultured DPCs for 2 days and (**C**) DPCs at the third passage cultured for 3 days; scale bar: 200 µm. (**D**) Crystal violet staining of DPCs in a clonal formation assay; scale bar: 500 µm. (**E**) Alizarin red staining showed mineralization nodule formation in DPCs after 5 days of osteo/odontogenic induction; scale bar: 200 µm. (**F**) Oil Red O staining showed lipid droplet formation in DPCs after 21 days of adipogenic induction; scale bar: 100 µm. (**G**) Flow cytometry results indicated that DPCs exhibited positive staining for MSC markers including CD29, CD44, and CD90 and negative staining for hematopoietic stem cell markers CD34 and CD45. (**H**) DPCs demonstrated positive expression for Vimentin and negative expression for Cytokeratin (top row); the negative control is presented on the bottom left; WSU-HN6 cells served as a positive control for Cytokeratin (bottom right); scale bar: 50 µm. (**I**) Relative mRNA levels of *Piezo1*, *Dspp*, *Dmp1*, and *Alp* of OS treated DPCs for 1, 3, 5, and 7 days. (**J**) Immunofluorescent staining of Piezo1 in DPCs showed that Piezo1 was expressed throughout the entire range of DPCs; scale bar: 50 µm. (**K**) Western blotting and (**L**) quantitative analysis of OS treated DPCs for 1, 3, 5, and 7 days showed that Piezo1, DSPP, and DMP1 protein levels significantly increased and mostly peaked on day 5. Compared with the NC group, * *p* < 0.05, ** *p* < 0.01, *** *p* < 0.001. PN, postnatal.

Next, we isolated, purified, and cultured DPCs according to our previous methods to perform further studies [24,25] (Figure 2A). DPCs migrated from the tissue blocks within 2 days, exhibiting a radial growth pattern with a dense center and a sparse periphery (Figure 2B). Other types of cells were scarcely detectable after multiple passages and purifications (Figure 2C). DPCs were capable of forming visible clones, and they were observed to have a polygonal, triangular, or spindle shape with broad cytoplasm and multiple protrusions (Figure 2D). The cells differentiated into osteo/odontogenic and adipogenic lineages under certain inducing conditions (Figure 2E,F). Flow cytometric analysis revealed that DPCs expressed the mesenchymal stem cell markers CD29, CD44, and CD90, while lacking expression of the hematopoietic stem cell markers CD34 and CD45 (Figure 2G). IF results indicated that DPCs were positively stained for Vimentin and negatively for Cytokeratin (Figure 2H). In summary, we successfully isolated DPCs and identified them as MSCs with multi-lineage differentiation potential.

Subsequently, we examined the pattern of Piezo1 in vitro. IF results revealed that Piezo1 was distributed throughout the entire range of DPCs (Figure 2J). Compared with regular culture conditions, the mRNA level of *Piezo1* in DPCs under the OS condition showed a similar upward trend in odontogenic differentiation markers such as *Dspp*, *Dmp1*, and *Alp*, mostly peaking on day 5 with statistically significant differences (Figure 2I). WB results showed a consistent trend in Piezo1, DSPP, and DMP1 protein expression (Figure 2K,L). These results suggest that Piezo1 is expressed in DPCs and is likely related to odontogenic differentiation and dentin formation in DPCs.

### 3.2. Knockdown of Piezo1 Inhibited Odontogenic Differentiation of DPCs

**Figure 3:**
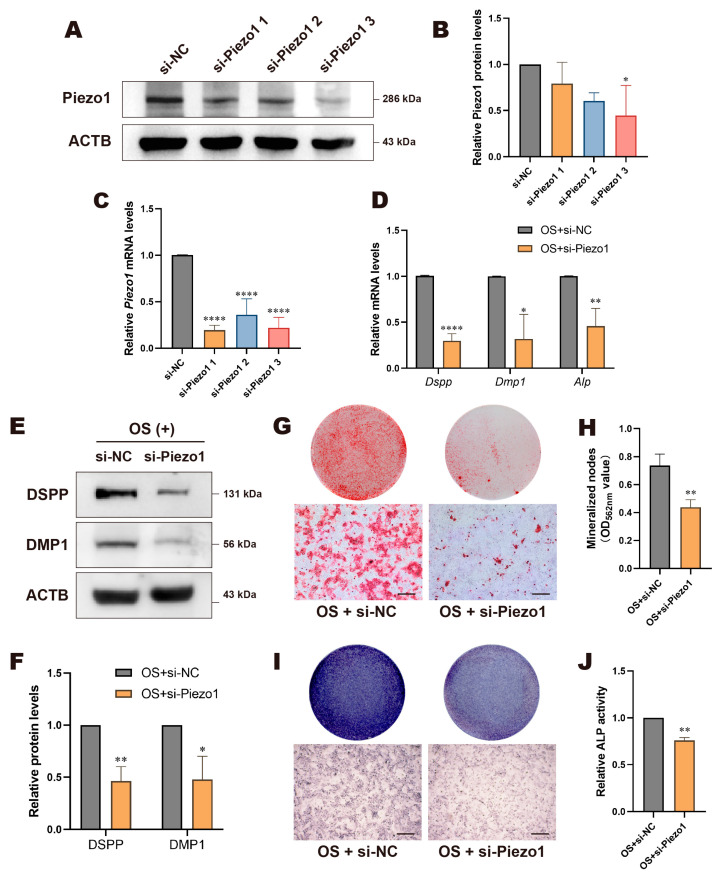
Knockdown of Piezo1 inhibited odontogenic differentiation of DPCs. Knockdown efficiency of Piezo1 was detected by (**A**) western blotting, (**B**) its quantitative analysis, and (**C**) RT-qPCR. (**D**) Knockdown of Piezo1 suppressed the relative mRNA expression of *Dspp*, *Dmp1*, and *Alp* in DPCs. (**E**) Western blotting and (**F**) quantitative analysis of DSPP and DMP1 protein levels in Piezo1-knockdown cells. (**G**) Alizarin red staining and (**H**) its semi-quantitative analysis revealed that knockdown of Piezo1 hindered mineralization nodule formation; scale bar: 500 µm. (**I**) ALP staining and (**J**) ALP activity decreased after Piezo1 knockdown; scale bar: 500 µm. Compared with si-NC or OS + si-NC group, * *p* < 0.05, ** *p* < 0.01, **** *p* < 0.0001.

We intervened in the activation state or expression level of Piezo1 to understand its role in the odontogenic differentiation process of DPCs. First, siRNA transfection was used to knock down Piezo1 in DPCs. The knockdown efficiency of mRNA and protein was assessed 48 h or 72 h post-transfection, respectively. As shown in Figure 3A–C, all three si-Piezo1 constructs effectively suppressed *Piezo1* mRNA expression, with si-Piezo1 3 showing the most significant decrease in protein expression. Thus, si-Piezo1 3 (hereafter referred to as si-Piezo1) was selected for subsequent experiments. The results showed that both mRNA and protein levels of odontogenic markers significantly decreased after Piezo1 knockdown (Figure 3D–F). Similarly, Piezo1 deficiency resulted in a significant reduction in mineralized nodules (Figure 3G,H), lighter ALP staining (Figure 3I), and a marked decrease in relative ALP activity (Figure 3J) in the si-Piezo1 group. These findings suggest that the knockdown of Piezo1 inhibits the odontogenic differentiation of DPCs.

### 3.3. Yoda1 Activation of Piezo1 Promoted Odontogenic Differentiation of DPCs

**Figure 4:**
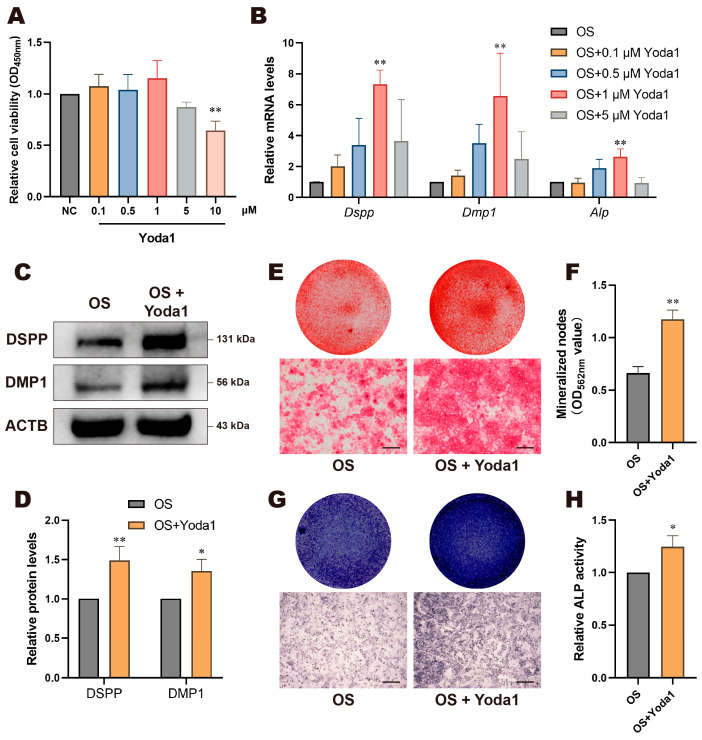
Yoda1 activation of Piezo1 promoted odontogenic differentiation of DPCs. (**A**) CCK-8 assay detected relative cell viability of DPCs treated with Yoda1 (0.1 µM, 0.5 µM, 1 µM, 5 µM, and 10 µM). (**B**) DPCs treated with Yoda1 upregulated the relative mRNA levels of *Dspp*, *Dmp1*, and *Alp*, with the 1 µM group showing greatest significance. (**C**) Western blotting and (**D**) quantitative analysis of DSPP and DMP1 protein levels of DPCs treated with Yoda1. (**E**) Alizarin red staining and (**F**) its semi-quantitative analysis revealed that Yoda1 promoted mineralization nodule formation; scale bar: 200 µm. (**G**) ALP staining and (**H**) ALP activity were enhanced by Yoda1; scale bar: 500 µm. Compared with NC or OS group, * *p* < 0.05, ** *p* < 0.01.

Next, cells were treated with Piezo1 agonist Yoda1. Given the cytotoxic effects of high concentrations of Yoda1 on cells [29], we performed a CCK-8 assay to determine the optimal concentration for treating DPCs. The results showed that Yoda1 concentrations below 1 μM had minimum effect on cell viability, whereas concentrations of 5 μM and above impaired cell viability, with the 10 μM group showing a statistically significant difference (Figure 4A). RT-qPCR results indicated that the addition of Yoda1 significantly increased the mRNA expression of *Dspp*, *Dmp1*, and *Alp*, with the 1 μM group showed greatest significance (Figure 4B). Based on these results and other studies [22,30,31], we selected 1 μM Yoda1 for subsequent experiments. Consistently, the protein expression levels of DSPP and DMP1 in the OS + Yoda1 group were upregulated significantly (Figure 4C,D). The OS + Yoda1 group showed more mineralized nodules in ARS (Figure 4E,F), deeper ALP staining (Figure 4G), and a significant increase in relative ALP activity (Figure 4H). These findings indicate that the activation of Piezo1 by Yoda1 promotes the odontogenic differentiation of DPCs, which corroborates the results of Piezo1 knockdown and demonstrates that Piezo1 is involved in regulating the odontogenic differentiation process of DPCs.

### 3.4. Activation of Piezo1 Channels Promoted Odontogenesis of DPCs in a 3D Culture Model and In Vivo

**Figure 5:**
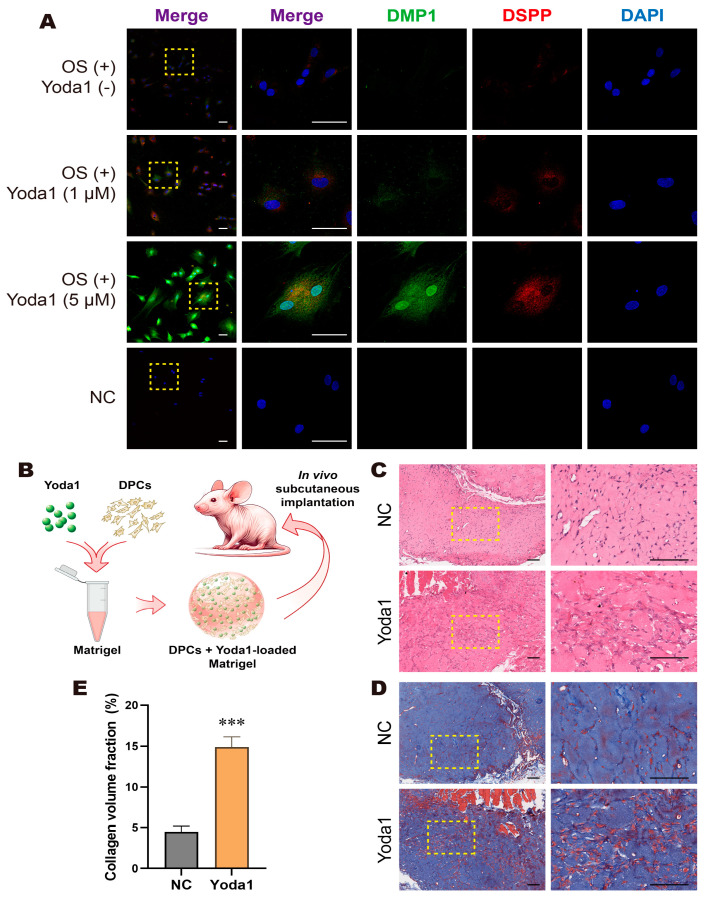
Activation of Piezo1 channels promoted odontogenesis of DPCs in a 3D culture model and in vivo. (**A**) Immunofluorescent staining results of the 3D culture model showed that Yoda1 treatment, especially in the 5 µM Yoda1 group, upregulated DMP1 (green) and DSPP (red) expression in DPCs; scale bar: 50 µm. (**B**) Schematic model of Matrigel seeded with DPCs and loaded with Yoda1. (**C**) H and E staining and (**D**) Masson trichrome staining revealed Yoda1 enhanced dentin and collagen formation in vivo; scale bar: 100 µm. (**E**) Quantitative analysis of collagen volume fraction. Compared with the NC group, *** *p* < 0.001.

We constructed a 3D culture model in which cells were cultured in Matrigel containing different concentrations of Yoda1. IF results showed that the fluorescence intensity of DSPP and DMP1 was upregulated in the Yoda1 treatment groups, with the 5 μM group showing a more obvious increase (Figure 5A). Based on these results, we constructed an ectopic odontogenesis model in Balb/c-nude mice in order to confirm Piezo1’s role in odontogenesis in vivo. We subcutaneously implanted Matrigel carrying DPCs with or without 5 μM Yoda1 on either side of the mouse (Figure 5B). After 4 weeks, the composites were removed and subjected to histological analysis. H and E staining (Figure 5C) and Masson trichrome staining (Figure 5D) results showed that the Yoda1 group had significantly more dentin collagen formation compared to the control group. Quantitative analysis of collagen formation based on Masson trichrome staining revealed that the collagen volume ratio in the Yoda1 group was significantly higher than that in the control group (Figure 5E). These results demonstrate that Piezo1 channel activation by Yoda1 promotes the dentin formation of DPCs in vivo.

### 3.5. FAM83G Negatively Regulated Piezo1-Mediated Odontogenic Differentiation of DPCs

**Figure 6:**
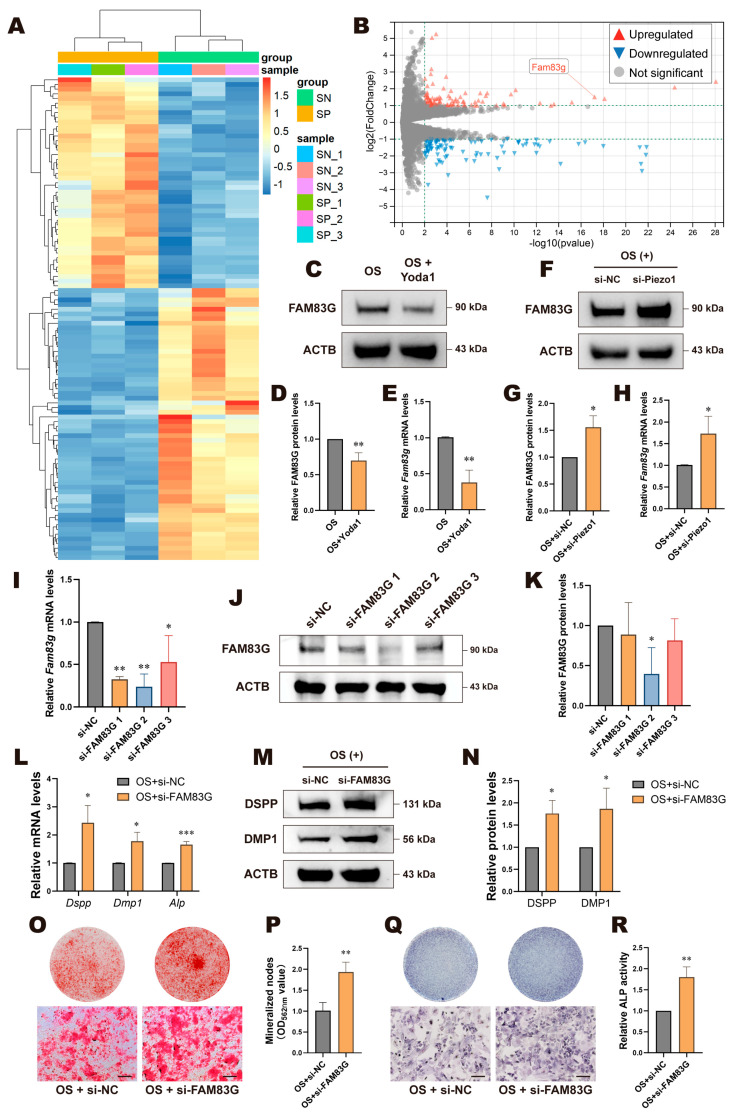
FAM83G negatively regulated Piezo1-mediated odontogenic differentiation of DPCs. (**A**) The heatmap of all DEGs and (**B**) the volcano plot of RNA-seq data comparing OS + si-NC to OS + si-Piezo1, where FAM83G is highlighted. (**C**) Western blotting and (**D**) quantitative analysis of FAM83G protein levels in Yoda1-treated cells. (**E**) Yoda1 inhibited the relative mRNA expression of *Fam83g*. (**F**) Western blotting and (**G**) quantitative analysis of FAM83G protein levels in Piezo1-knockdown cells. (**H**) Knockdown of Piezo1 enhanced the relative mRNA expression levels of *Fam83g*. Knockdown efficiency of FAM83G was detected using (**I**) RT-qPCR, (**J**) western blotting, and (**K**) quantitative analysis. (**L**) Knockdown of FAM83G upregulated the expression of *Dspp*, *Dmp1*, and *Alp* at the mRNA level. (**M**) Western blotting and (**N**) quantitative analysis of DSPP and DMP1 protein levels in FAM83G-knockdown cells. (**O**) Alizarin red staining and (**P**) its semi-quantitative analysis revealed that knockdown of FAM83G promoted mineralization nodule formation; scale bar: 500 µm. (**Q**) ALP staining and (**R**) ALP activity increased after FAM83G knockdown; scale bar: 200 µm. Compared with si-NC or OS + si-NC group, * *p* < 0.05, ** *p* < 0.01, *** *p* < 0.001. SN, OS + si-NC; SP, OS + si-Piezo1.

To further investigate how Piezo1 regulates the odontogenic differentiation of DPCs, we employed RNA-seq to compare the OS + si-Piezo1 group with the OS + si-NC group, identifying DEGs in the transcriptome, which included 45 upregulated and 58 downregulated candidate genes (Figure 6A,B and Table S1). After excluding downstream genes previously associated with Piezo1 and validating the results using multiple RT-qPCR experiments, our attention turned to FAM83G, an upregulated gene. Current research on FAM83G is sparse, and no studies have addressed its role in dentin formation. We conducted RT-qPCR and WB validation in the OS + Yoda1 and OS + si-Piezo1 groups. The results were consistent with RNA-seq, indicating that when Piezo1 was knocked down, the expression of FAM83G was upregulated. Conversely, when cells were treated with Yoda1, FAM83G was downregulated (Figure 6C–H). Subsequently, we performed knockdown of FAM83G with siRNA and verified the efficacy of si-FAM83G 2 (hereafter referred to as si-FAM83G) (Figure 6I–K). RT-qPCR and WB results revealed all odontogenic markers were upregulated in FAM83G-knockdown cells (Figure 6L–N). Likewise, knockdown of FAM83G led to increased mineralized nodules in ARS (Figure 6O,P), as well as elevated ALP activity (Figure 6Q,R). These results suggest that FAM83G may be a downstream gene regulated by Piezo1 during odontogenic differentiation of DPCs and act as a negative regulator in this process.

## 4. Discussion

Our study demonstrated that Piezo1 plays a critical role in promoting odontogenic differentiation of DPCs, as evidenced by both in vitro pharmacological activation and Piezo1 knockdown, as well as in vivo experiments. DPCs are a population of MSCs that play a pivotal role in tooth development and regeneration. They are precursor cells for odontoblasts and dental pulp cells, playing a critical role in tooth morphogenesis as well as pulp and dentin formation. They exhibit strong self-renewal capacity and multi-lineage differentiation potential [32]. Previous studies have demonstrated that pathways such as TGF-β [33,34], Wnt [35], mitochondrial function [24,25], and WW domain-containing E3 ubiquitin-protein ligase 2 [36,37] are essential in regulating the odontogenic differentiation of DPCs. Due to these characteristics, DPCs are considered ideal seed cells for studying tooth development and dental tissue engineering. Further investigations into DPCs offer new perspectives for dental tissue engineering and hold potential for developing cell-based therapeutic strategies to address tooth defects and diseases [24,38].

Mechanical stimulation is a significant factor influencing tooth development. Some evidence suggests that orthodontic forces may promote early-stage dentin formation in immature permanent teeth [1,3]. However, research on the effects of mechanical forces, particularly mechanosensitive ion channels, on tooth development remains scarce. To address this gap, we focused on Piezo1, a mechanosensitive ion channel belonging to the Piezo family. Piezo proteins are evolutionarily conserved transmembrane proteins present in animals, plants, and protozoa, but absent in yeast and bacteria [7]. In mammals, the Piezo family comprises two homologs, namely, Piezo1 and Piezo2, which share high sequence homology and structural similarity [7]. Functionally, Piezo1 is predominantly expressed in non-sensory tissues and plays a pivotal role in sensing mechanical stimuli (e.g., membrane tension and hydrostatic pressure), while Piezo2 is primarily localized to sensory neurons and specialized mechanoreceptors such as dorsal root ganglia and Merkel cells [9,10,11]. Given these tissue-specific expression patterns, our study focuses on Piezo1 rather than Piezo2. In addition to Piezo1, other transmembrane proteins are also emerging as key players in the odontogenic differentiation of DPCs. For example, the absence of transmembrane protein 30B (TMEM30B) has been shown to impair odontoblastic differentiation in mouse DPCs, reducing both intracellular and secreted protein levels [39]. Syndecan-1, the expression of which correlates closely with cell proliferation and differentiation, may promote the odontogenic differentiation of DPCs by modulating interactions between the extracellular matrix and signaling molecules [40]. Previous studies have shown that Piezo1 is involved in osteogenesis and odontogenesis in stem cells via pathways like Wnt/β-catenin [14,23], NF-κB [41], MAPK [42], and Akt [21] pathways. Other reports have highlighted the significant role of Piezo1 in promoting immune responses in macrophages and neutrophils. Therefore, the development of antibacterial dental materials based on Piezo1 is also a promising topic [43,44,45]. Generally, our findings extend this knowledge by specifically confirming Piezo1’s role in the odontogenic differentiation of DPCs, thus shedding light on its potential contribution to tooth development.

Studies have indicated that Piezo1 expression is upregulated under certain mechanical stimuli, accompanied by an increase in calcium ion influx [26,46]. In OTM, Piezo1 expression is upregulated on the tension side and downregulated on the compression side, contributing to bone remodeling [19,47]. Similarly, Yoda1, a specific Piezo1 agonist, acts as a wedge to force Piezo1 channel activation and induces calcium influx [27,48]. Although our experiments did not directly apply mechanical stimuli, the changes in Piezo1 expression and the pharmacological activation we employed mimicked mechanical stimulation conditions for DPCs. Furthermore, subsequent experiments confirmed Piezo1’s role in odontogenic differentiation. Thus, these findings reveal a previously underexplored aspect of mechanotransduction in dental tissue formation, offering both theoretical bases and critical insights into the effects of OTM on immature permanent teeth.

In our study, we observed that Piezo1 was distributed both in the cytoplasm and on the nuclear membrane of DPCs without the application of external mechanical force. This distribution pattern aligns with the overall cell contour. Previous studies have similarly shown that Piezo1 is not only localized on the plasma membrane [7] but also on other subcellular structures, such as the nuclear membrane [49] and the endoplasmic reticulum [50]. Moreover, it has been reported that Piezo1’s subcellular localization changes dynamically depending on the mechanical environment. In the absence of mechanical stimuli, Piezo1 primarily resides in the nuclear membrane or is dispersed centrally within the cell, whereas upon compression, it forms large cytoplasmic clusters and eventually relocates to the nuclear membrane to facilitate processes like cell extrusion [49,51]. Our findings extend these studies, further supporting the notion that Piezo1’s subcellular localization is influenced by both cellular and environmental contexts.

We explored the effects of Yoda1, a specific agonist of Piezo1, on DPCs odontogenic differentiation. Our results demonstrated that Yoda1, at concentrations between 0.5 and 5 μM, upregulated the mRNA expression of odontogenic markers in DPCs, with the 1 μM concentration yielding the most significant effect. These findings align with previous studies that have reported similar effective concentrations for Yoda1 in in vitro cell culture [22,23,30,31]. Additionally, we observed that high concentrations of Yoda1 exerted cytotoxic effects, a result consistent with earlier research [29] and confirmed by our experiment. Interestingly, despite its potent activation of Piezo1 channels, Yoda1 does not seem to alter Piezo1 expression levels [28], a finding we also verified in this study using RT-qPCR analysis (Figure S1). Moreover, our IF results from 3D culture in Matrigel indicated that 5 μM of Yoda1 was more effective in inducing odontogenic differentiation in this environment. This concentration was subsequently applied to the experiment in nude mice, where it similarly confirmed Yoda1’s efficacy in promoting odontogenic differentiation in vivo. Our findings suggest that Yoda1 could be used as a potential therapeutic agent for dentin regeneration or as a component of dental cavity base materials to promote dentin formation. However, the specific mechanisms through which Yoda1 influences these processes remain to be elucidated.

We uncovered FAM83G as a novel regulator of Piezo1-mediated odontogenic differentiation in DPCs. Using RNA-seq analysis, we found that Piezo1 knockdown resulted in a significant upregulation of FAM83G expression. To our knowledge, this appears to be the inaugural investigation to correlate Piezo1 with FAM83G. Subsequent experiments confirmed that suppressing FAM83G expression promoted odontogenic differentiation of DPCs, implying that FAM83G plays a role in maintaining cell stemness. FAM83G has been implicated in various pathways, including BMP and Wnt signaling [52,53]. FAM83G is primarily expressed in the cytoplasm, with detectable levels also found in the nucleus, cell membrane, and cytoskeleton [52,54]. Similar to Piezo1, its subcellular localization changes under different conditions [54]. For example, FAM83G undergoes nuclear translocation upon BMP stimulation. In the context of cancer, FAM83G is highly expressed in tumor tissues, promoting cell proliferation, migration, and invasion, leading to poor prognosis [55,56,57]. This supports our hypothesis regarding its role in maintaining stemness. Our findings are particularly intriguing as they suggest that FAM83G negatively regulates Piezo1-mediated odontogenesis, a role not previously identified. Additionally, our study raises the possibility that FAM83G may play a role in maintaining stemness, which warrants further investigation. Further studies are required to elucidate the detailed mechanisms by which FAM83G influences DPC differentiation and how Piezo1 and FAM83G interact to better understand their broader implications in tooth development.

## 5. Conclusions

Collectively, this study identifies DPCs as MSCs with multi-lineage differentiation potential and demonstrates that Piezo1 expression increases during the odontoblastic differentiation of DPCs both in vivo and in vitro. By activating Piezo1 with Yoda1 and knocking down Piezo1, we confirm that Piezo1 regulates the odontogenic differentiation of DPCs in vitro and in vivo. Using RNA-seq analysis and subsequent screening, we identify that FAM83G plays a negative regulatory role in Piezo1-mediated odontogenic differentiation, a role not previously reported in this area of research. This work offers new insights into the mechanisms of tooth development and dentin formation, highlighting Piezo1 as a potential target for dental tissue regeneration.

## Figures and Tables

**Table 1:** Antibodies.

Antibodies	Dilution	Purchased From
anti-Cytokeratin primary antibody	1:100 for IF	Boster Bio, Wuhan, Hubei, China (PB9715)
anti-Vimentin primary antibody	1:100 for IF	Boster Bio, Wuhan, Hubei, China (PB9359)
anti-Piezo1 primary antibody	1:500 for WB 1:50 for IHC/IF	Proteintech, Rosemont, IL, USA (15939-1-AP)
anti-DSPP primary antibody *	1:500 for WB 1:200 for IF	Santa Cruz, Santa Cruz, CA, USA (LFMb-21)
anti-DMP1 primary antibody **	1:1000 for WB 1:200 for IF	Affinity, Lexington, MA, USA (DF8825)
anti-ACTB primary antibody	1:1000 for WB	Affinity, Lexington, MA, USA (AF7018)
anti-FAM83G primary antibody	1:500 for WB	Invitrogen, Carlsbad, CA, USA (PA5-54775)
Dylight 488/594 conjugated secondary antibody	1:200	EarthOx, Burlingame, CA, USA (E032220, E032410)
HRP-conjugated secondary antibodies	1:1000	Beyotime, Shanghai, China (A0208, A0350)
FITC anti-CD44-IgG2a antibody	5 μL/test	Elabscience, Wuhan, Hubei, China (E-AB-F1225C)
FITC anti-CD45-IgG1 antibody	5 μL/test	Elabscience, Wuhan, Hubei, China (E-AB-F1227C)
FITC anti-CD90-IgG1 antibody	5 μL/test	Elabscience, Wuhan, Hubei, China (E-AB-F1226C)
PE anti-CD29-IgG antibody	5 μL/test	Elabscience, Wuhan, Hubei, China (E-AB-F1309D)
PE anti-CD34-IgG2a antibody	5 μL/test	Elabscience, Wuhan, Hubei, China (E-AB-F1284UD)

* DSPP, dentin sialophosphoprotein. ** DMP1, dentin matrix protein 1.

**Table 2:** RT-qPCR primers.

Gene	Forward Primer (5′-3′)	Reverse Primer (5′-3′)
*Piezo1*	TTGCGTACGTTCACGAAGGA	TTCGCTCACGTAAAGCTGGT
*Dspp*	ACAGCGACAGCGACGATTC	CCTCCTACGGCTATCGACTC
*Dmp1*	ACCAAAATACTGAATCTGAAAGCTC	TGCTGTCCGTGTGGTCACTA
*Alp* *	GGAAGGAGGCAGGATTGA	TCAGCAGTAACCACAGTCA
*Fam83g*	CGTACAGGTCAATCTGGGGG	GGGAGAGGGGTCTCGTTTCT
*Actb*	CGGTCAGGTCATCACTATC	CAGCACTGTGTTGGCATA

* *Alp*, alkaline phosphatase.

**Table 3:** siRNA sequences of Piezo1 and FAM83G.

Gene	Forward Primer (5′-3′)	Reverse Primer (5′-3′)
si-NC	UUCUCCGAACGUGUCACGUTT	ACGUGACACGUUCGGAGAATT
si-Piezo1 1	UGGCGCCGGCCAUCUUGUUUGUUUA	UAAACAAACAAGAUGGCCGGCGCCA
si-Piezo1 2	UGGUACUUCGUGAAGUGCAUUUACU	AGUAAAUGCACUUCACGAAGUACCA
si-Piezo1 3	CCGUCAUCAUCUCUAAGAAUAUGUU	AACAUAUUCUUAGAGAUGAUGACGG
si-FAM83G 1	CAGGAGCUGUACCUUAUGUTT	ACAUAAGGUACAGCUCCUGTT
si-FAM83G 2	GGGCCAACAUGUUUGAGUATT	UACUCAAACAUGUUGGCCCTT
si-FAM83G 3	CAGGGACUCACAGGAUAUATT	UAUAUCCUGUGAGUCCCUGTT

## Data Availability

The original contributions presented in this study are included in the article/Supplementary Material. Further inquiries can be directed to the corresponding authors.

## Supplementary Materials

The following supporting information can be downloaded at: https://www.mdpi.com/article/10.3390/biom15030316/s1, Figure S1: The effect of Yoda1 on *Piezo1* expression at the mRNA level was not statistically significant; Table S1: DEGs identified using RNA-seq. File S1: Western Blotting Figures.

## Abbreviations

The following abbreviations are used in this manuscript:

ACTB	β-Actin
ALP	Alkaline phosphatase
ARS	Alizarin red staining
BCA	Bicinchoninic acid
DEGs	Differentially expressed genes
DMP1	Dentin matrix protein 1
DPCs	Dental papilla cells
DSPP	Dentin sialophosphoprotein
ECL	Enhanced chemiluminescence
FAM83G	Family with sequence similarity 83, member G
FBS	Fetal bovine serum
H and E	Hematoxylin and eosin
IF	Immunofluorescence
IHC	Immunohistochemistry
mOB	Mature odontoblasts
MSCs	Mesenchymal stem cells
NC	Negative control
OD	Optical density
OS	Osteo/Odontogenic induction
OTM	Orthodontic tooth movement
PMSF	Phenylmethylsulfonyl fluoride
pOB	Preodontoblasts
PVDF	Polyvinylidene fluoride
RIPA	Radioimmunoprecipitation assay
RNA-seq	RNA sequencing
RT-qPCR	Real-time quantitative polymerase chain reaction
SD	Sprague-Dawley
WB	Western blotting
