# Supplementary material for: Piezo1 Regulates Odontogenesis via a FAM83G-Mediated Mechanism in Dental Papilla Cells In Vitro and In Vivo
Source: Biomolecules. 2025 Feb 20;15(3):316. doi: 10.3390/biom15030316 (PMC11940480; doi:10.3390/biom15030316)
Supplement: Supplementary file 1 [file biomolecules-15-00316-s001.zip › Table S1.pdf]

Table S1. DEGs of RNA-seq.

Gene	log2(FoldChange)	p-value	change
Cxcl3	2.430143192	1.06E-24	UP
Cldn1	2.090653	2.59E-21	UP
Mmp13	-1.951644651	4.82E-19	DOWN
Sod3	-1.487351495	4.82E-19	DOWN
Nsun4	-2.9136608	8.70E-19	DOWN
Megf6	-2.491343194	8.87E-19	DOWN
Piezo1	-1.504842181	9.76E-19	DOWN
Cd38	1.41003989	1.22E-15	UP
Tspan18	-1.426726515	1.39E-15	DOWN
Fam83g	1.513657495	7.56E-15	UP
Dkk2	-1.204892689	4.44E-14	DOWN
Apoe	-1.30277387	1.56E-13	DOWN
Sema3e	1.199529254	5.85E-13	UP
Mepe	-2.487487294	5.60E-12	DOWN
Syde1	1.090031602	2.06E-11	UP
Mfsd6	1.004544203	3.58E-11	UP
C1s	-1.152628445	4.36E-11	DOWN
Flvcr2	1.154837231	3.05E-10	UP
Crlf1	-1.442388517	3.10E-10	DOWN
Ptn	-1.342984354	4.58E-10	DOWN
Akr1c14	-1.834046739	5.31E-10	DOWN
Igfbp2	-1.038344636	5.31E-10	DOWN
Ptx3	-1.32304802	7.40E-10	DOWN
Megf10	-1.117633165	3.45E-09	DOWN
Spon1	-1.314987361	3.70E-09	DOWN
Cntfr	-1.390264751	6.82E-09	DOWN
Col6a3	-1.191138572	1.56E-08	DOWN
Lbp	-1.174460965	1.56E-08	DOWN
Aard	-1.230725396	1.58E-08	DOWN
Des	1.931548172	2.65E-08	UP
Ifitm1	-1.849407882	3.30E-08	DOWN
Tspan4	1.683322573	6.60E-08	UP
Nts	-1.010061435	1.33E-07	DOWN
Bmp6	1.03197654	2.21E-07	UP
Hnrnpab	-1.818331615	2.44E-07	DOWN
Dusp2	-1.150426594	2.51E-07	DOWN
Tlk1	1.119823333	2.56E-07	UP
Ogn	-1.454001551	3.34E-07	DOWN
Ankrd24	-2.808691209	5.68E-07	DOWN
Eln	-2.136701955	8.24E-07	DOWN
Fn1	1.01842546	9.29E-07	UP
Fgf1	-1.051238726	2.34E-06	DOWN

Gdpd2	-1.910179322	3.90E-06	DOWN
Aspn	-4.497969094	4.65E-06	DOWN
Tnxb	1.748121108	4.65E-06	UP
Csmd1	1.587605914	6.10E-06	UP
Dapk1	1.247779623	1.14E-05	UP
LOC691995	-1.152375307	1.40E-05	DOWN
Rcsd1	-1.924390805	1.54E-05	DOWN
Nefm	1.745089959	1.64E-05	UP
Tuba8	1.310683587	2.32E-05	UP
Ptprz1	-1.026721661	2.38E-05	DOWN
Kcnk2	-1.840447869	0.0000305	DOWN
Atp6v0e2	1.011970985	0.0000305	UP
Ramp1	-1.9963517	0.0000354	DOWN
Tmem246	-1.19151796	0.0000428	DOWN
Msln	1.217889754	0.0000428	UP
Sgms2	1.016549785	0.0000436	UP
LOC100911440	-2.321889788	0.0000474	DOWN
Ablim3	1.049477849	0.0000821	UP
Tnfrsf19	-1.190417403	0.0000838	DOWN
MGC109340	-1.017569978	0.000102887	DOWN
Tmeff2	-2.261309403	0.000214576	DOWN
Lpo	1.755845336	0.000242044	UP
Cpeb2	1.016824285	0.00025649	UP
Pcdh1	1.042573041	0.000288812	UP
Plekha7	1.249214654	0.000297541	UP
RGD1566029	-1.767634542	0.00034058	DOWN
Pvrl4	1.235028463	0.00034058	UP
Dsp	1.521086075	0.00054028	UP
Grb14	1.055812144	0.000810921	UP
Igsf9b	1.275033154	0.000810921	UP
Scg2	1.392555152	0.001169836	UP
Lynx1	1.260039624	0.001247102	UP
Srl	1.11502919	0.001777951	UP
Tfap2c	-1.040970537	0.001794479	DOWN
Capn8	1.022174447	0.001809653	UP
Fras1	1.284510264	0.001945495	UP
Rmnd5b	-1.557420074	0.00208985	DOWN
Cnrip1	-1.058449629	0.002414431	DOWN
Slc7a11	1.079661743	0.002463595	UP
Ereg	1.885564773	0.002479762	UP
Egflam	-1.515778546	0.002642298	DOWN
Cd55	1.047376948	0.002796586	UP
Odam	1.455931628	0.002803581	UP
Map2k6	-1.091852577	0.003095394	DOWN

Scube2	1.588732014	0.003612708	UP
Mif	-3.160286032	0.003993671	DOWN
Kcnc3	1.04406391	0.004191561	UP
Nol8	-2.017823354	0.004733603	DOWN
Wnt7a	1.046664643	0.004733603	UP
Pycard	-1.390960884	0.005339025	DOWN
LOC100910646	2.726330231	0.005445106	UP
Ak5	-1.122386168	0.005897219	DOWN
Rbm39	-1.606825873	0.005952107	DOWN
Lyz2	-2.097865876	0.006054237	DOWN
Vstm2b	-1.69601795	0.006889593	DOWN
Myl10	-1.116324869	0.007284029	DOWN
Vsnl1	-1.077689977	0.007284029	DOWN
Kcnh1	1.025028437	0.007836052	UP
Cftr	2.030024596	0.007984785	UP
Dpep1	-1.064861391	0.009383112	DOWN
LOC681193	-2.420253605	0.009771755	DOWN
