# Supplementary figures and images for: Piezo1 Regulates Odontogenesis via a FAM83G-Mediated Mechanism in Dental Papilla Cells In Vitro and In Vivo
Source: Biomolecules. 2025 Feb 20;15(3):316. doi: 10.3390/biom15030316 (PMC11940480; doi:10.3390/biom15030316)

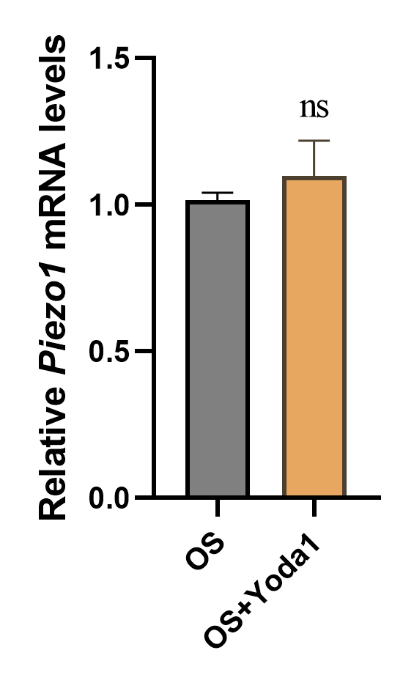

Supplement: Supplementary file 1 [file biomolecules-15-00316-s001.zip › Figure S1.png]

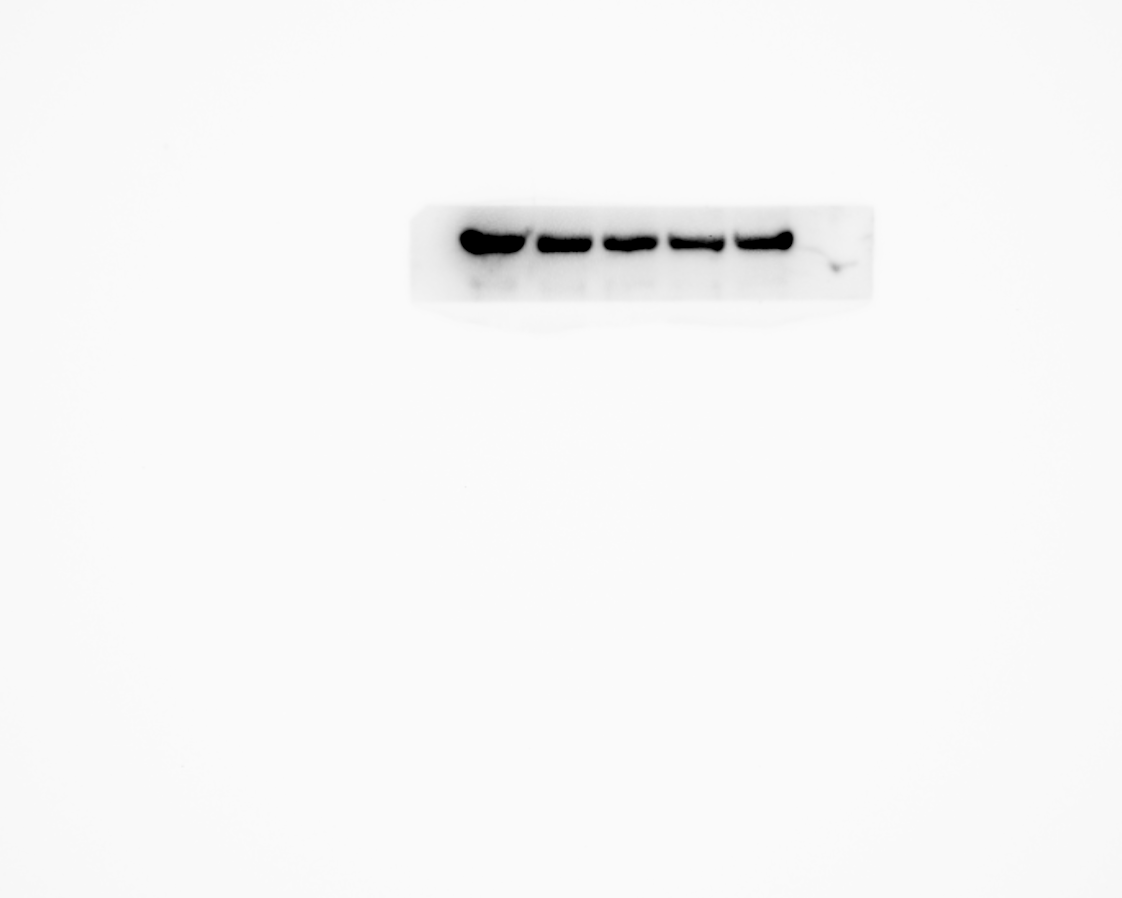

Supplement: Supplementary file 1 [file biomolecules-15-00316-s001.zip › File S1.Original blots/Figure 2K/Figure 2K ACTB.tif]

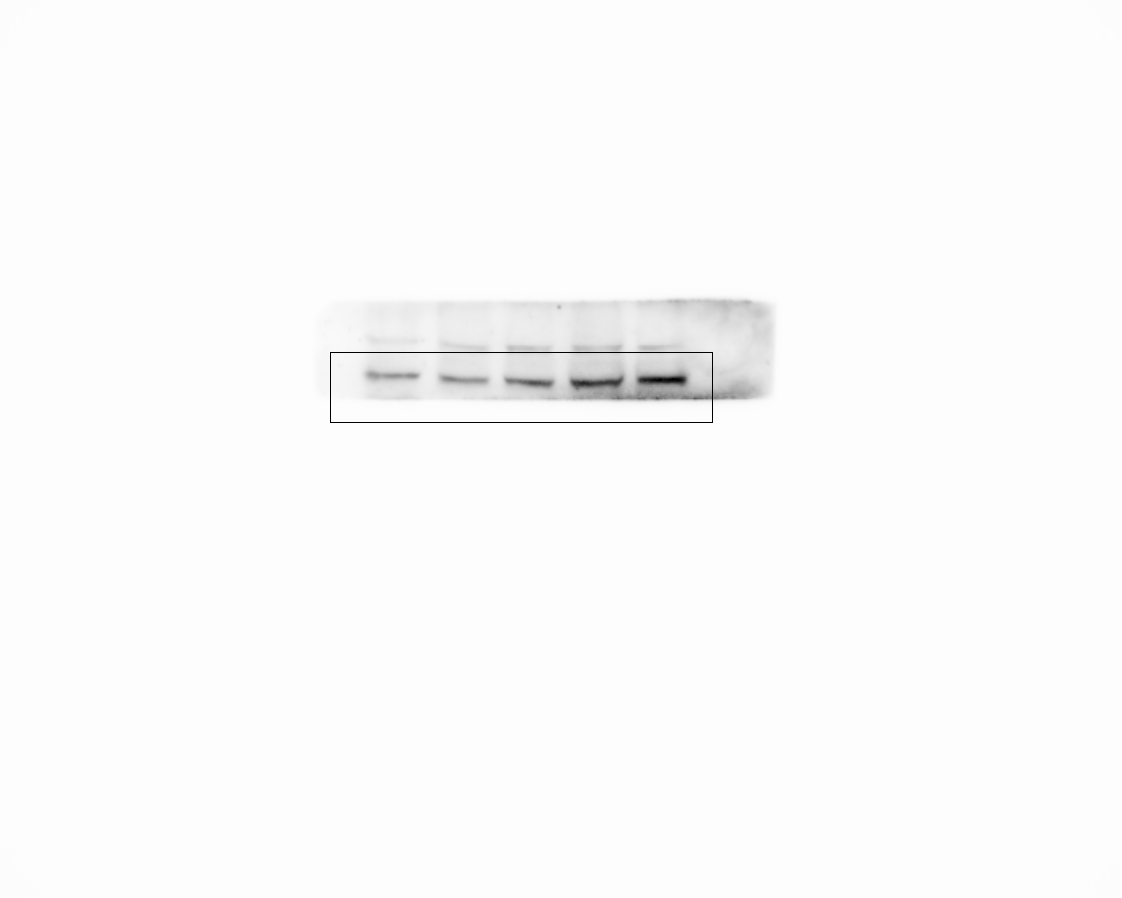

Supplement: Supplementary file 1 [file biomolecules-15-00316-s001.zip › File S1.Original blots/Figure 2K/Figure 2K DMP1.tif]

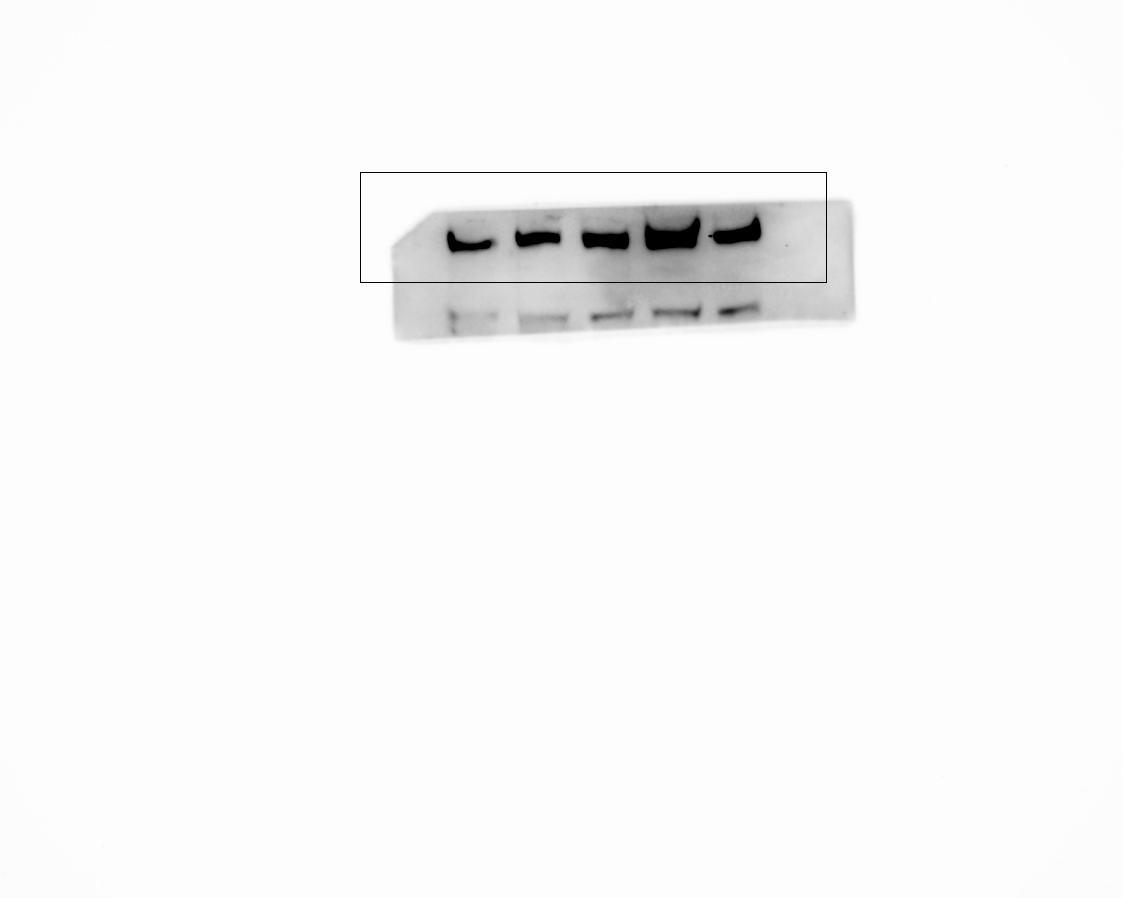

Supplement: Supplementary file 1 [file biomolecules-15-00316-s001.zip › File S1.Original blots/Figure 2K/Figure 2K DSPP.tif]

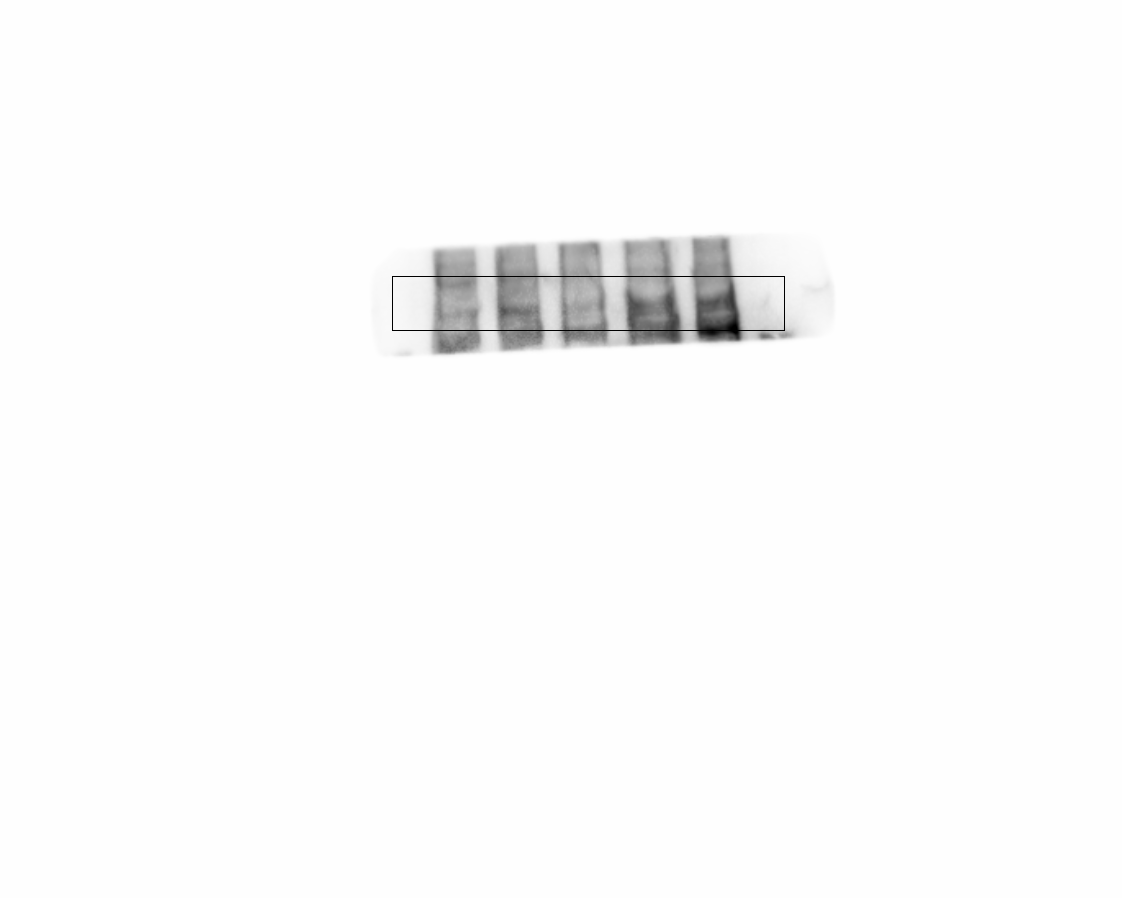

Supplement: Supplementary file 1 [file biomolecules-15-00316-s001.zip › File S1.Original blots/Figure 2K/Figure 2K Piezo1.tif]

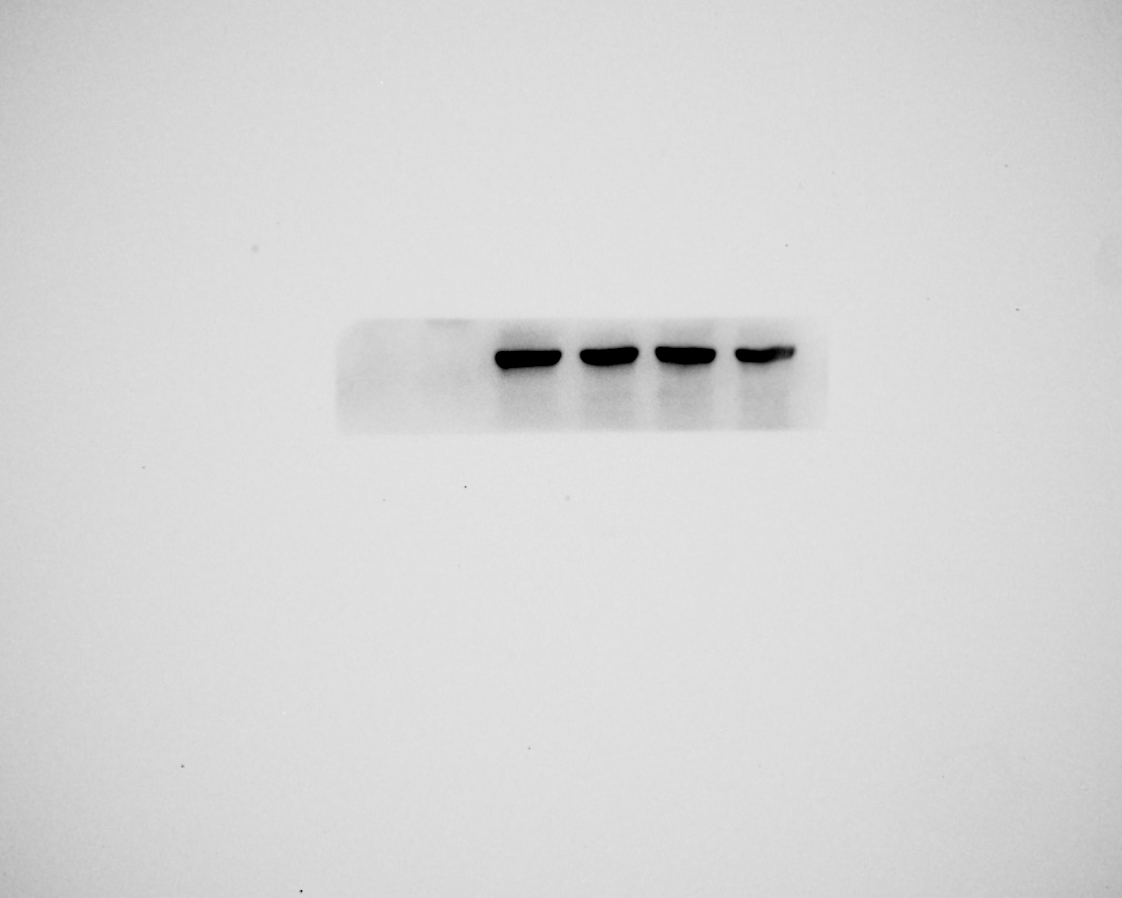

Supplement: Supplementary file 1 [file biomolecules-15-00316-s001.zip › File S1.Original blots/Figure 3A/Figure 3A ACTB.tif]

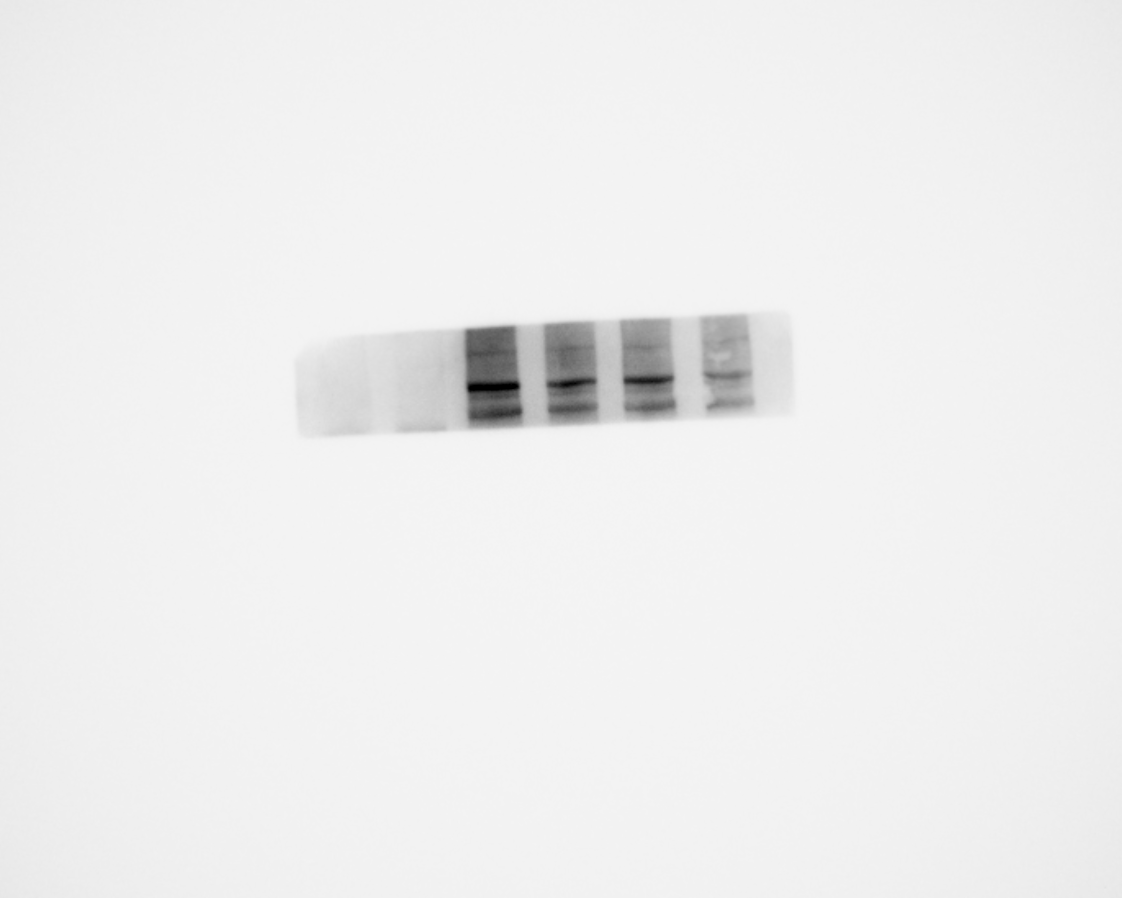

Supplement: Supplementary file 1 [file biomolecules-15-00316-s001.zip › File S1.Original blots/Figure 3A/Figure 3A Piezo1.tif]

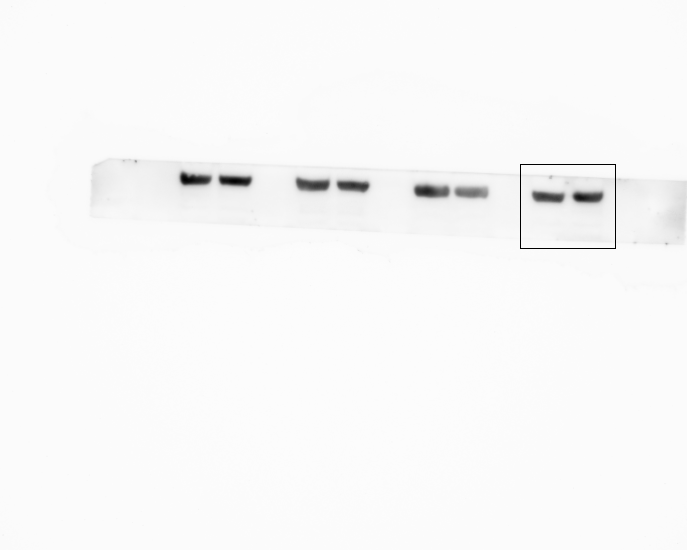

Supplement: Supplementary file 1 [file biomolecules-15-00316-s001.zip › File S1.Original blots/Figure 3E/Figure 3E ACTB.tif]

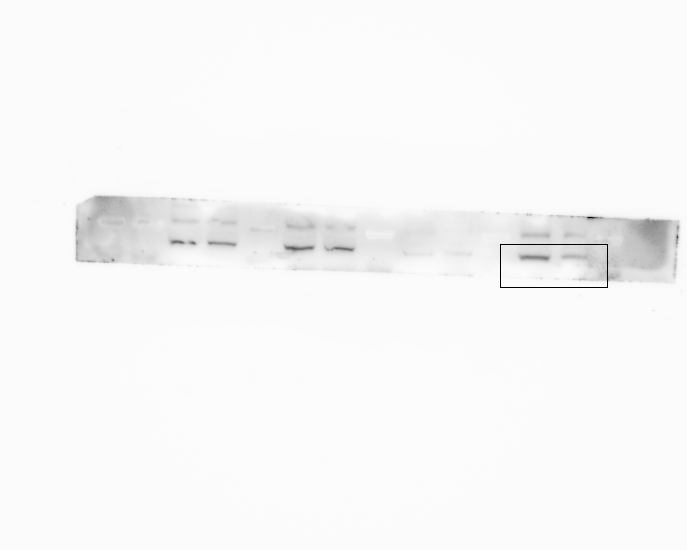

Supplement: Supplementary file 1 [file biomolecules-15-00316-s001.zip › File S1.Original blots/Figure 3E/Figure 3E DMP1.tif]

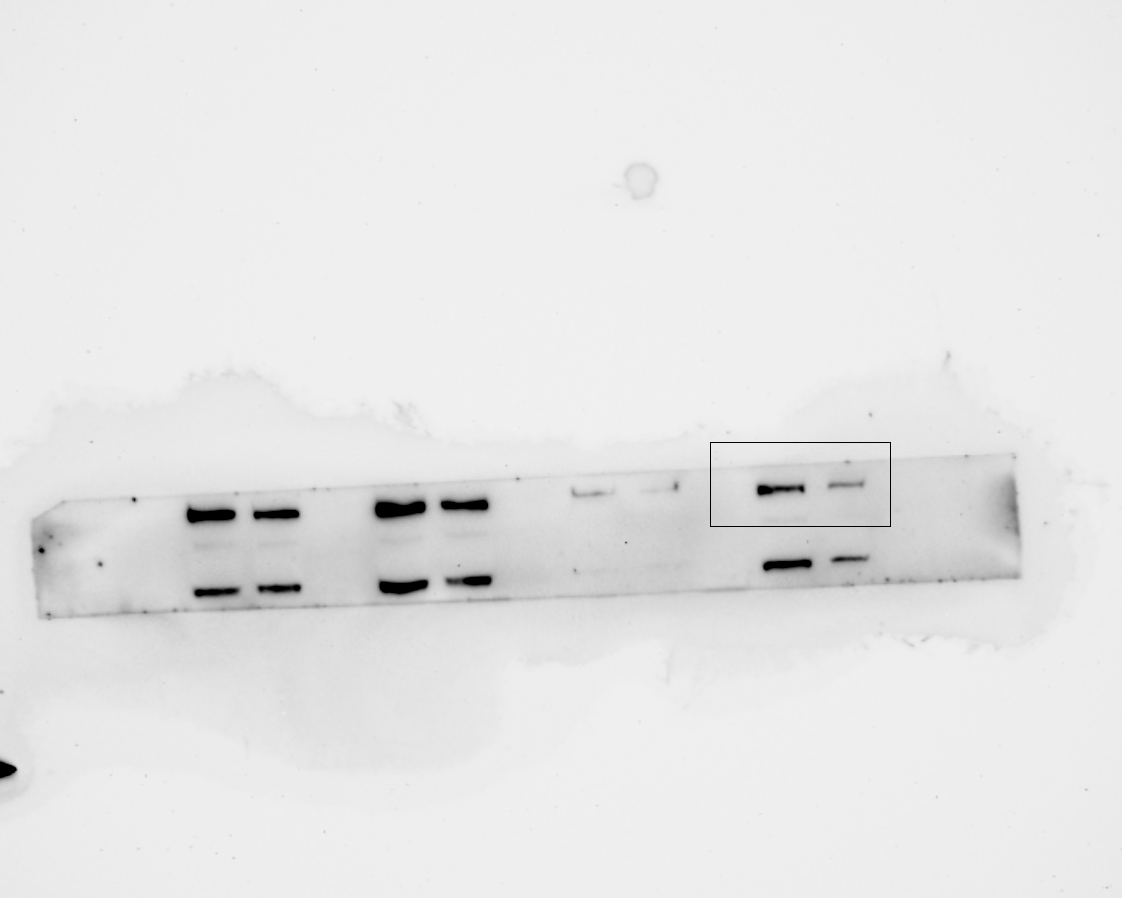

Supplement: Supplementary file 1 [file biomolecules-15-00316-s001.zip › File S1.Original blots/Figure 3E/Figure 3E DSPP.tif]

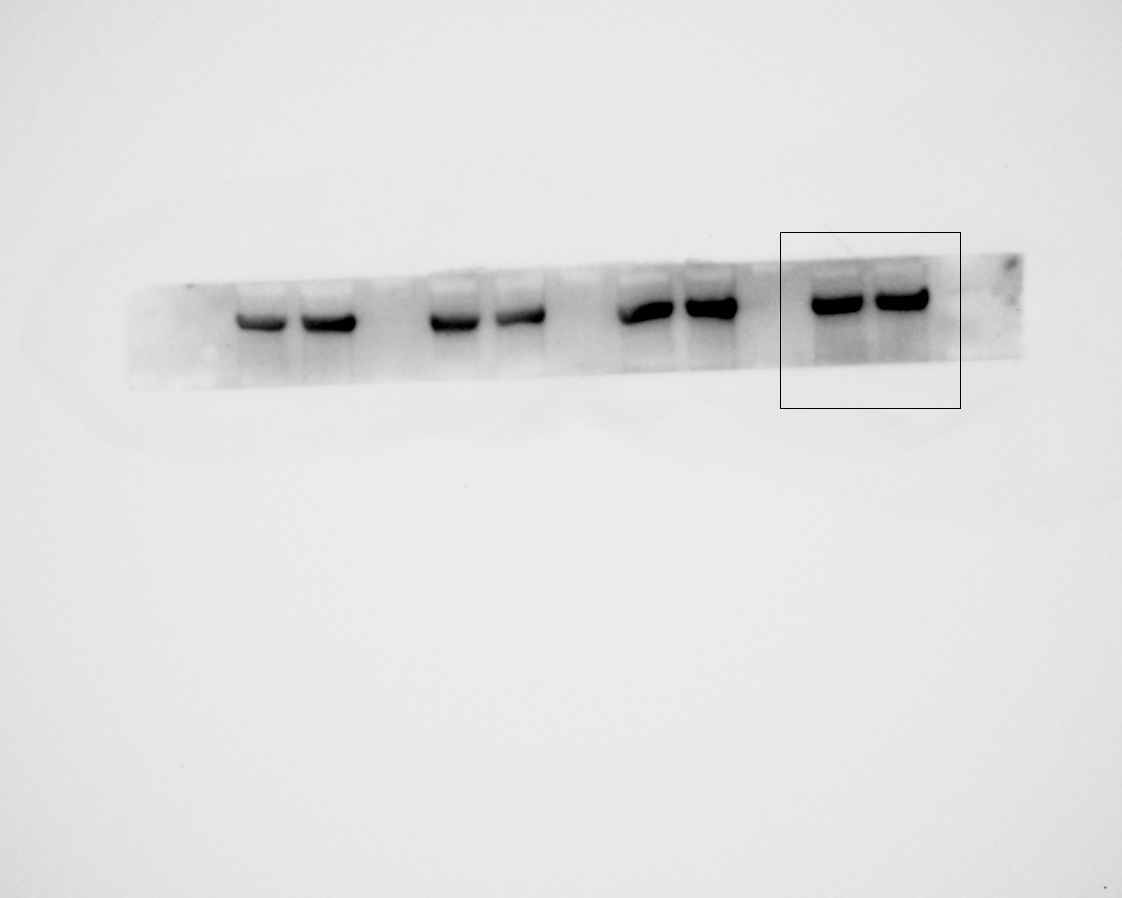

Supplement: Supplementary file 1 [file biomolecules-15-00316-s001.zip › File S1.Original blots/Figure 4C/Figure 4C ACTB.tif]

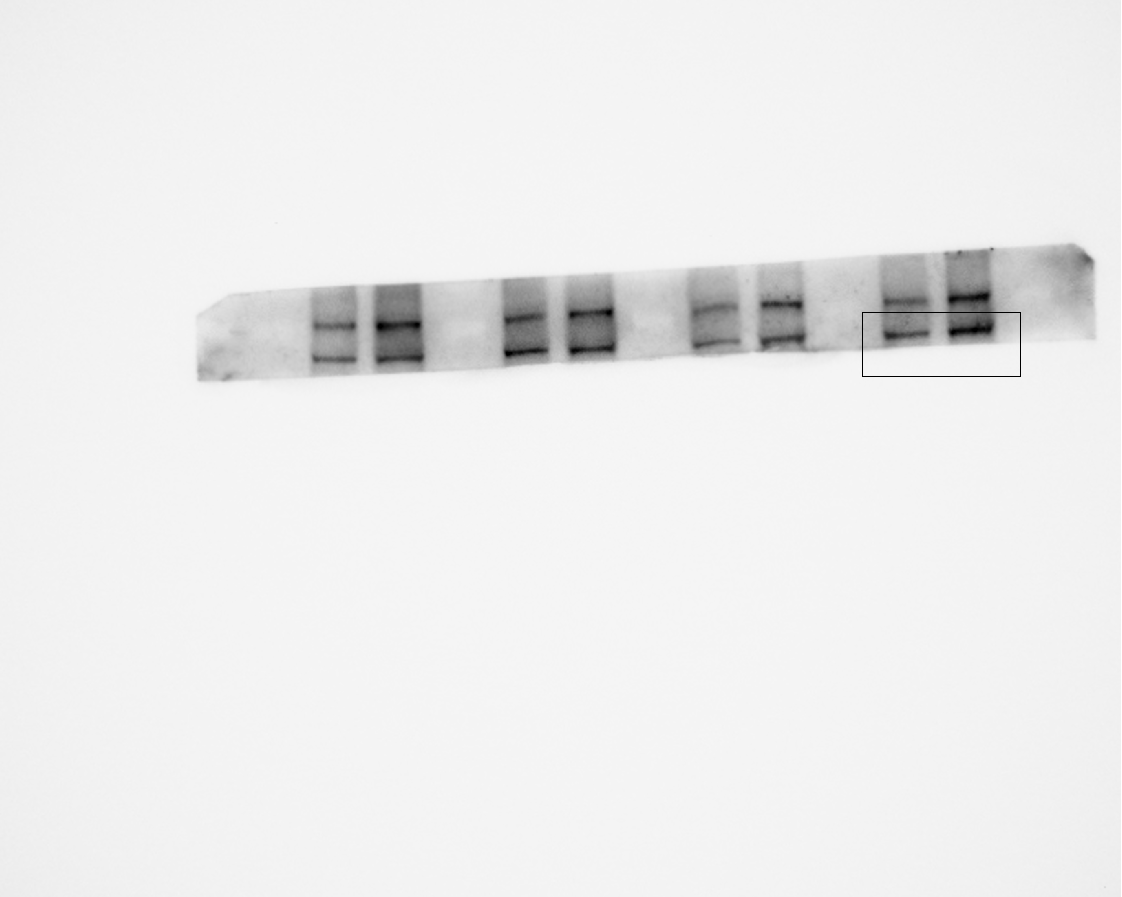

Supplement: Supplementary file 1 [file biomolecules-15-00316-s001.zip › File S1.Original blots/Figure 4C/Figure 4C DMP1.tif]

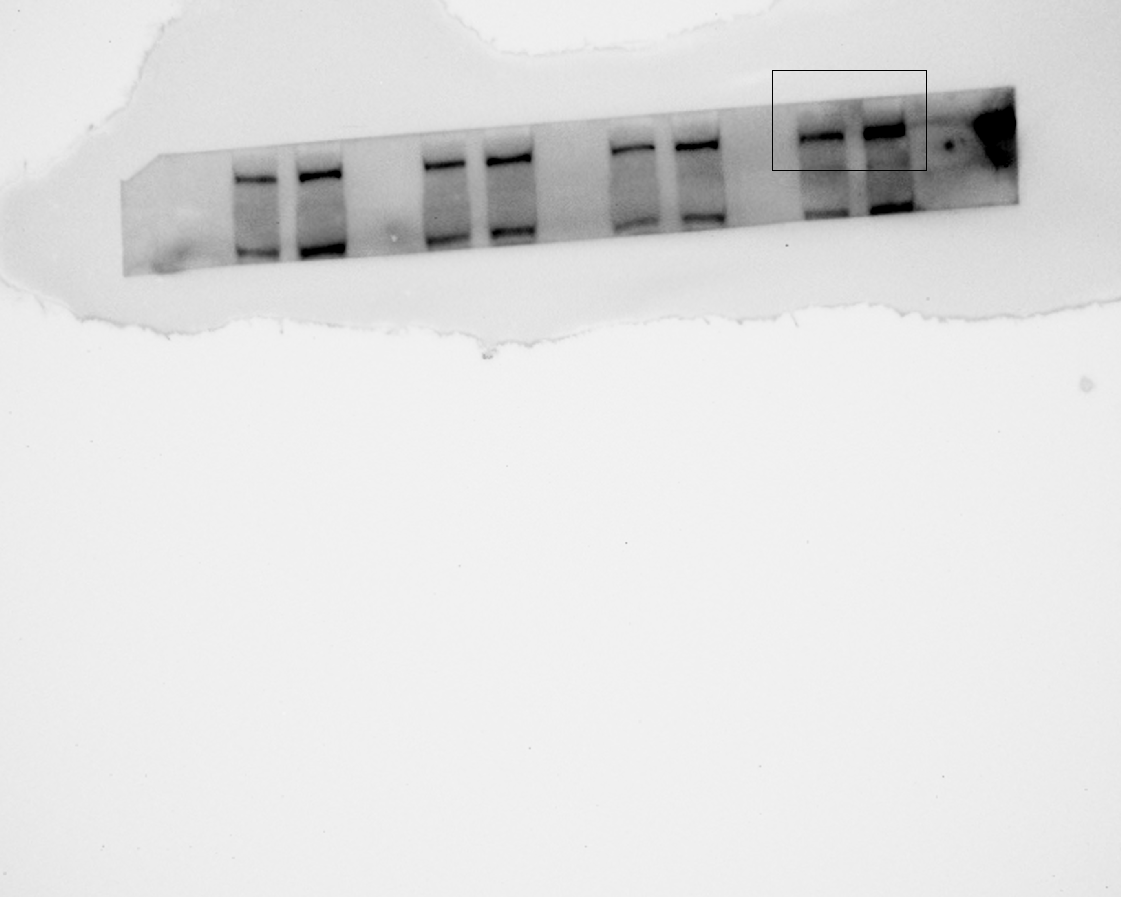

Supplement: Supplementary file 1 [file biomolecules-15-00316-s001.zip › File S1.Original blots/Figure 4C/Figure 4C DSPP.tif]

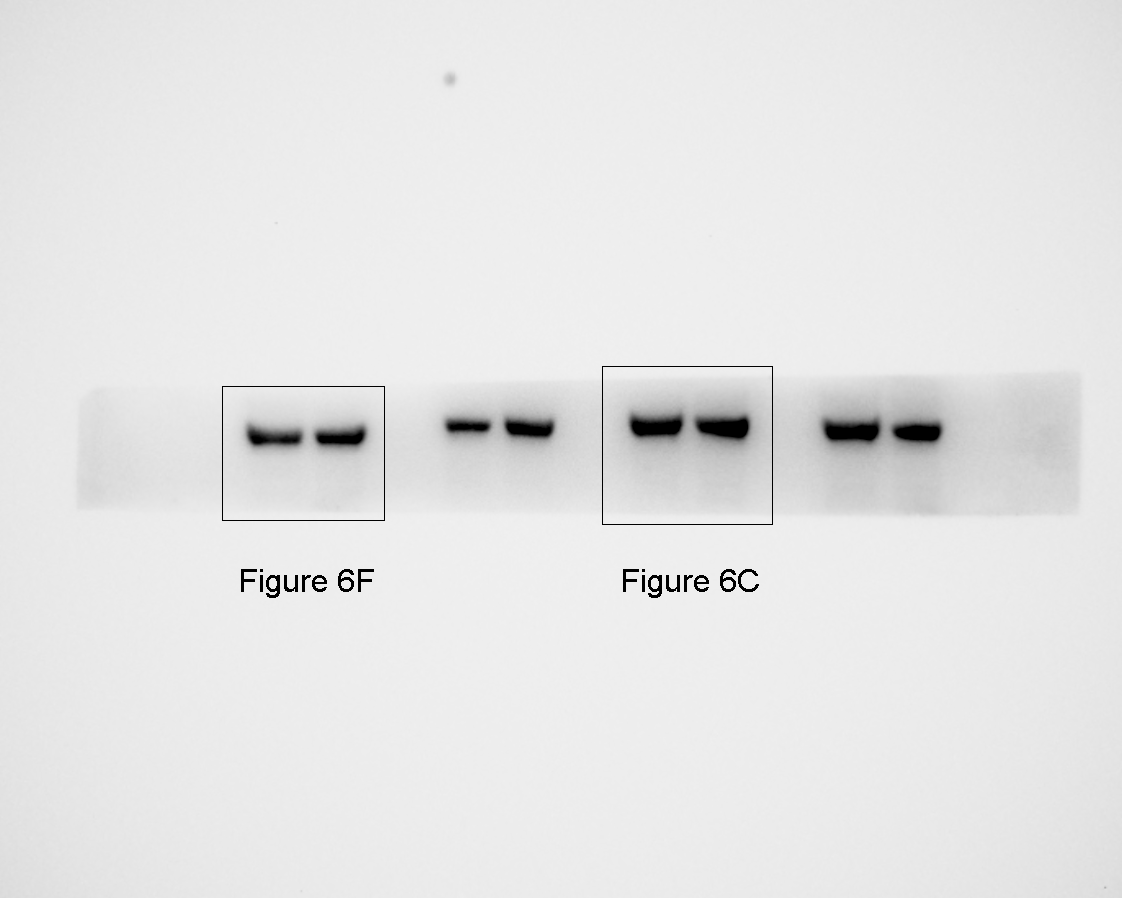

Supplement: Supplementary file 1 [file biomolecules-15-00316-s001.zip › File S1.Original blots/Figure 6C,F/Figure 6C,F ACTB.tif]

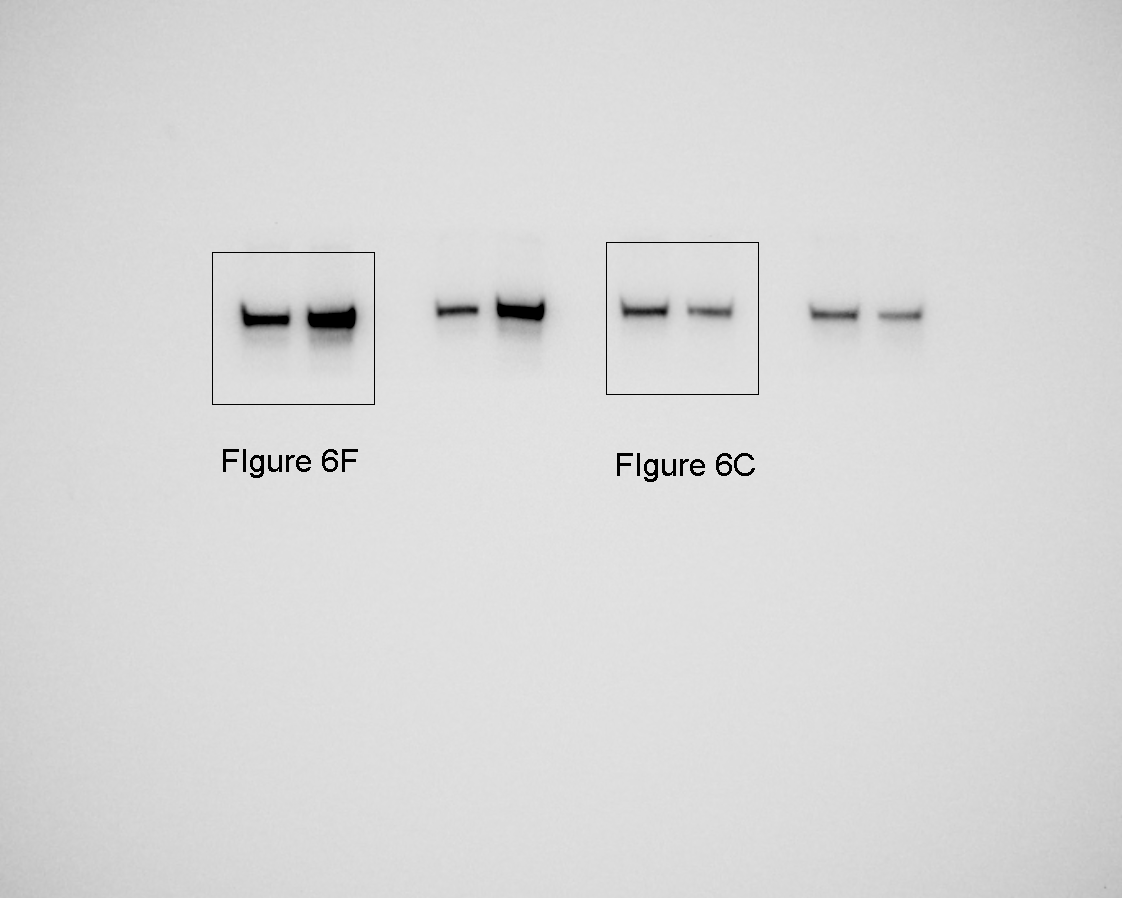

Supplement: Supplementary file 1 [file biomolecules-15-00316-s001.zip › File S1.Original blots/Figure 6C,F/Figure 6C,F FAM83G.tif]

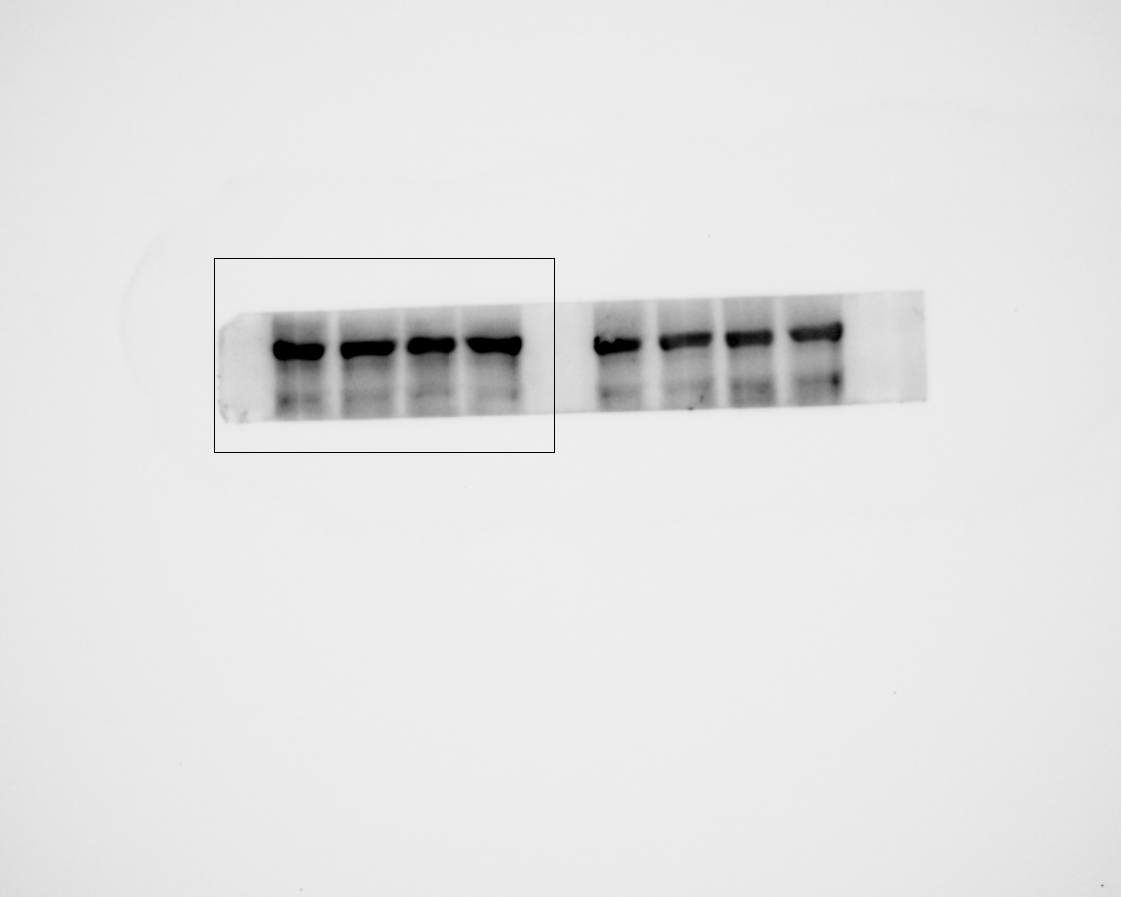

Supplement: Supplementary file 1 [file biomolecules-15-00316-s001.zip › File S1.Original blots/Figure 6J/Figure 6J ACTB.tif]

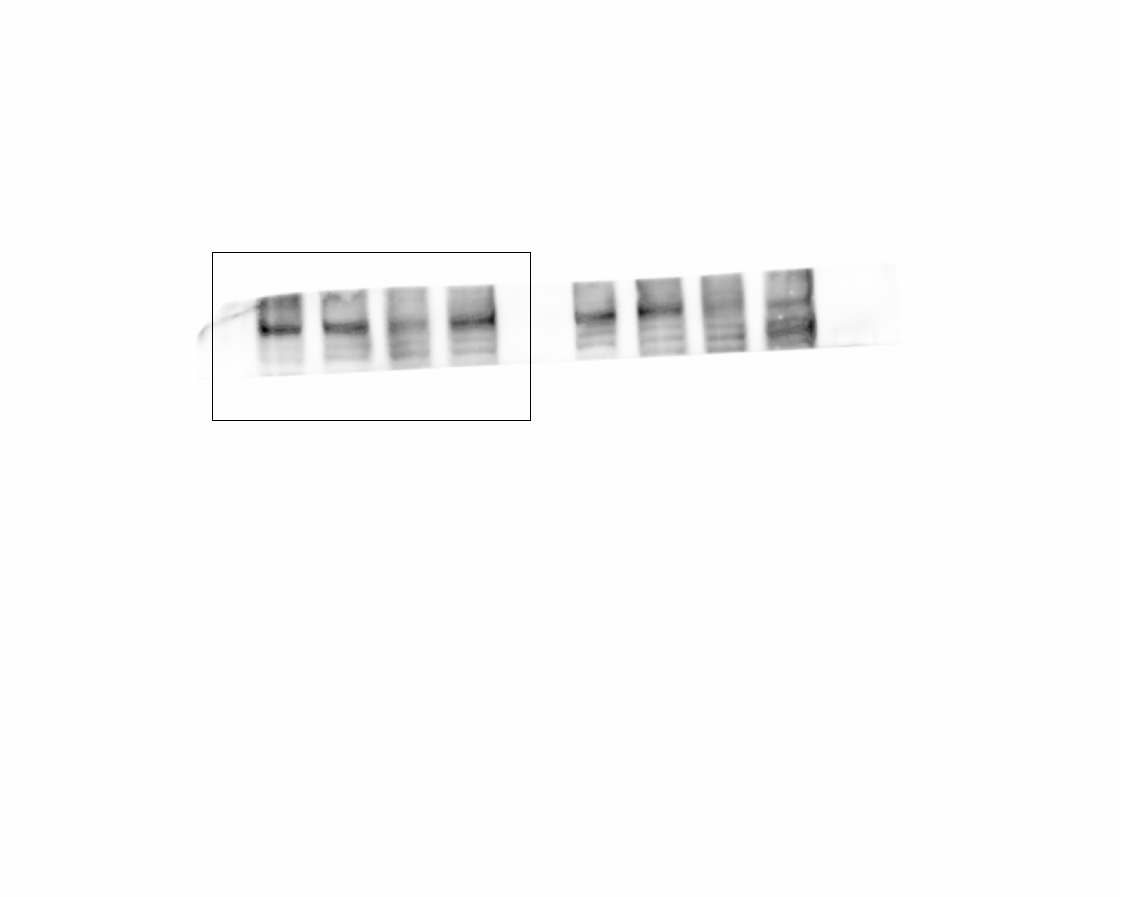

Supplement: Supplementary file 1 [file biomolecules-15-00316-s001.zip › File S1.Original blots/Figure 6J/Figure 6J FAM83G.tif]

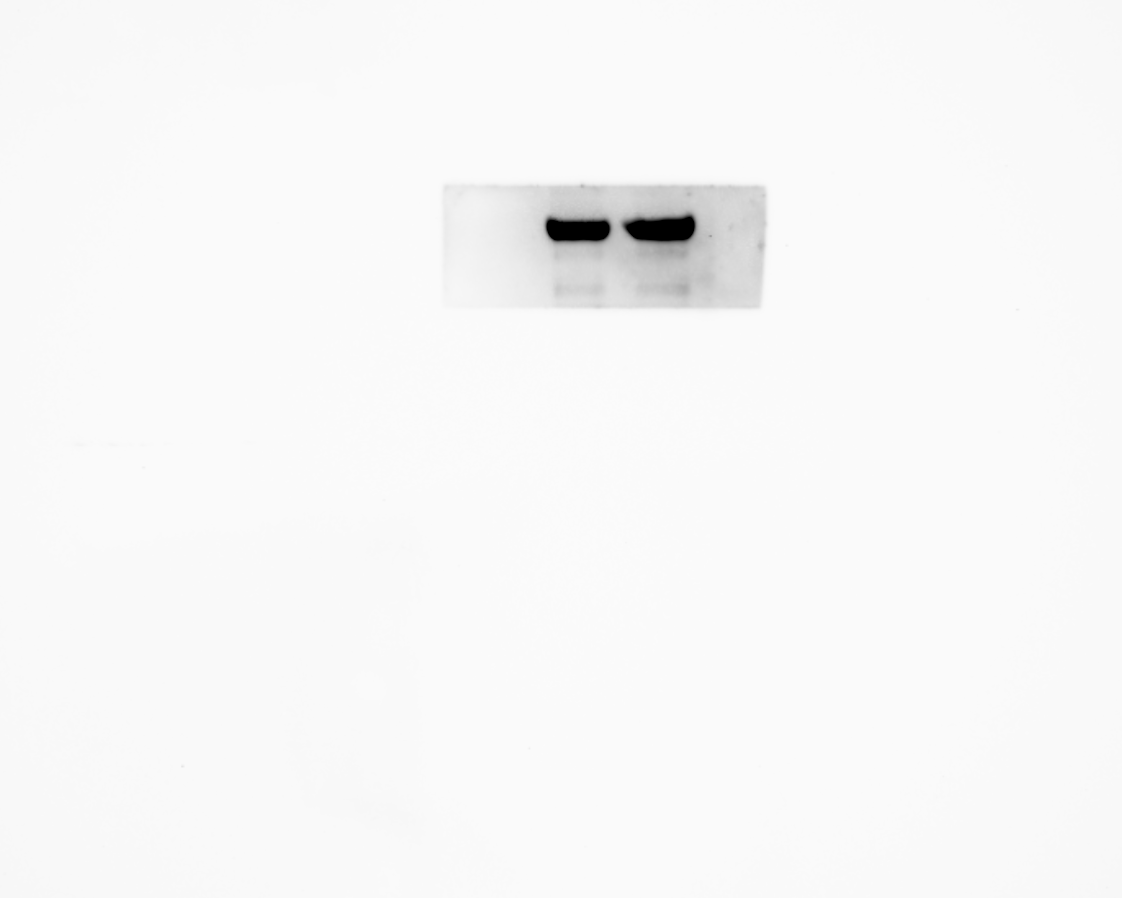

Supplement: Supplementary file 1 [file biomolecules-15-00316-s001.zip › File S1.Original blots/Figure 6M/Figure 6M ACTB.tif]

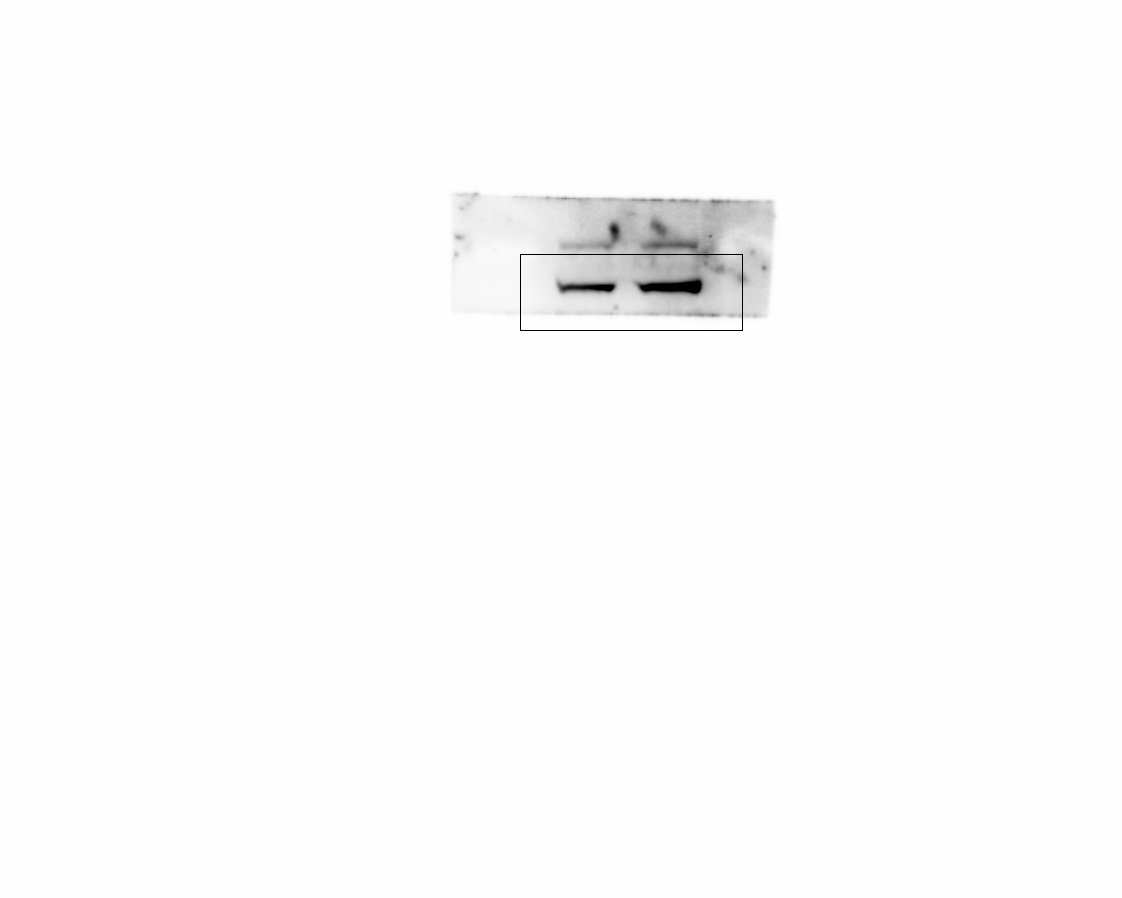

Supplement: Supplementary file 1 [file biomolecules-15-00316-s001.zip › File S1.Original blots/Figure 6M/Figure 6M DMP1.tif]

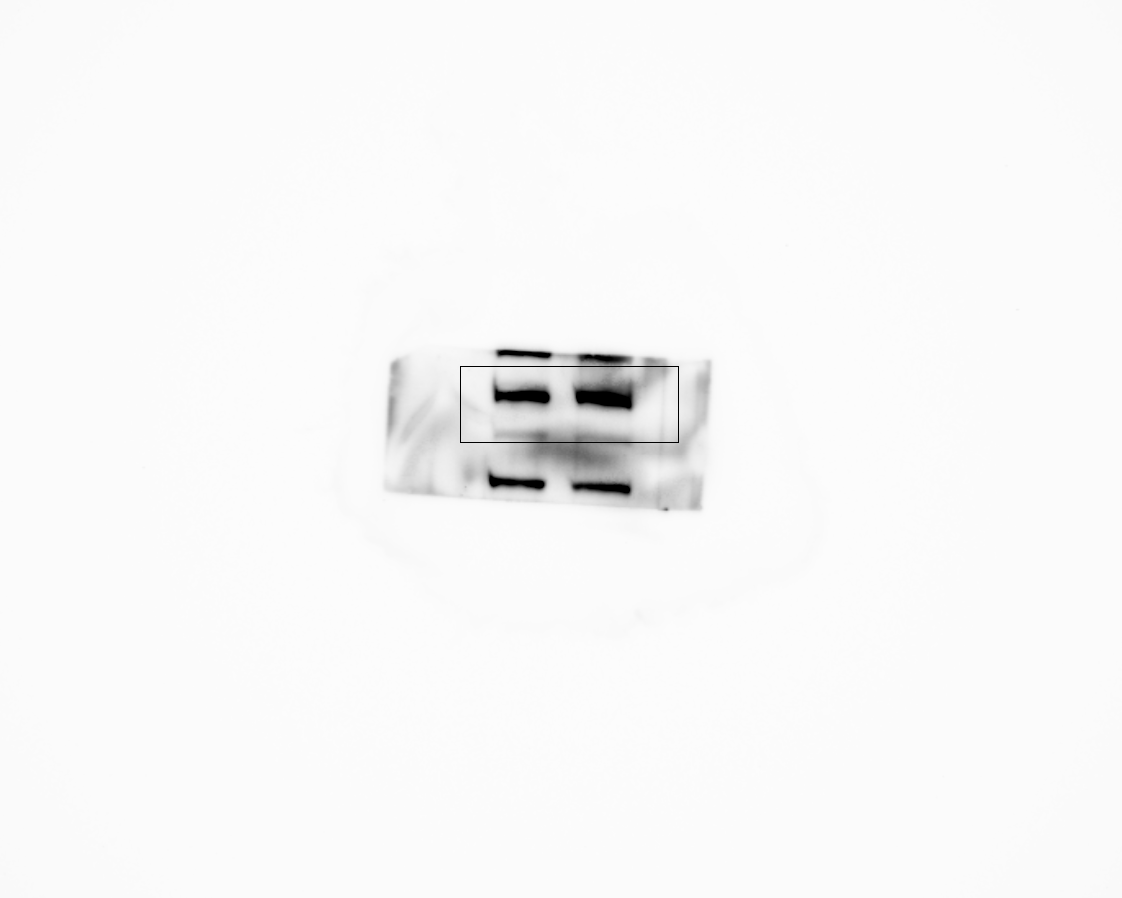

Supplement: Supplementary file 1 [file biomolecules-15-00316-s001.zip › File S1.Original blots/Figure 6M/Figure 6M DSPP.tif]
